# Brief Report: State Policy Contexts and Disability Risks Among Midlife Working-Age Latino Adults in the U.S.: Variation by Nativity and Citizenship Status

**DOI:** 10.1007/s11113-026-10022-6

**Published:** 2026-06-23

**Authors:** Marc A. Garcia, Blakelee R. Kemp, Catherine García, Courtney E. Boen, Rogelio Sáenz

**Affiliations:** 1 Department of Sociology, Aging Studies Institute, Syracuse University, Syracuse, NY USA; 2 Department of Child, Youth and Family Studies, University of Nebraska, Lincoln, NE USA; 3 Department of Human Development and Family Science, Syracuse University, Syracuse, NY USA; 4 Department of Sociology, Population Studies & Training Center, Brown University, Providence, RI USA; 5 Department of Sociology and Demography, University of Texas, San Antinio, TX USA

**Keywords:** Latino health; U.S. state policies; health disparities

## Abstract

Disability risks among midlife Latino adults in the United States are strongly patterned along axes of stratification such as nativity and citizenship status. Another increasingly salient axis of inequality in disability risk is geographic context, such as U.S. state political environments. While prior research highlights the importance of specific state policies, such as Medicaid, in shaping health-related outcomes, few studies have examined how broader policy contexts and ideologies, such as policy liberalism, influence disability risk, particularly among Latino adults. This study combines data from the 2008–2019 American Community Survey (ACS) with state-level measures of overall, economic, and social policy liberalism to examine how these dimensions of policy context influence disability risks among a diverse sample of midlife working-age Latino adults (*n* = 1,103,993). Using logistic regression models, we evaluate the associations between state policy liberalism (overall, economic, and social domains) and disability and assess differences by nativity and citizenship status. Results indicate that greater overall and economic policy liberalism were associated with lower probabilities of disability among midlife Latino adults. The magnitude of this association varied by nativity and citizenship, with U.S.-born Latino adults experiencing greater declines in disability across more liberal policy contexts compared to foreign-born Latino adults. Additionally, compared to the naturalized and non-citizens, citizens experienced greater declines in disability across more liberal policy contexts. These results suggest that state policy contexts that promote and protect social and economic supports, such as improving access to labor protections, can reduce the burden of disability, particularly among U.S.-born and citizen Latino adults.

**Supplementary Information:**

The online version contains supplementary material available at 10.1007/s11113-026-10022-6.

## Introduction and Background

Disability risks among midlife to older Latino adults in the United States are strongly patterned along axes of stratification, such as nativity and citizenship status (Burns et al., 2025; Garcia et al., 2019; Gubernskaya et al., 2013; Hayward et al., 2014; Patti & Boen, 2018). Another increasingly salient, but largely understudied, axis of inequality in Latino disability patterns is geographic context (Garcia & García, 2025; Kindratt & Smith, 2024), including state political environments (Montez & Grumbach, 2023). A substantial body of research demonstrates the significant impact of state policies, particularly those governing Medicaid, on outcomes such as mental health and access to healthcare. Emerging research suggests that U.S. states’ political and policy contexts are key social determinants of population health, in large part because they can either mitigate or exacerbate health inequalities (Montez & Grumbach, 2023; Montez et al., 2017), including among immigrants (Patti & Boen, 2018; Philbin et al., 2018; Schut & Boen, 2022; Wallace et al., 2019).

More liberal state policy contexts can help protect against health decline by expanding eligibility for economic support and social service benefits, both of which are associated with better long-term health and disability outcomes (Boen & Parrish, 2026). In contrast, more conservative policies can restrict access to essential public services, education, and labor protections, thereby contributing to the widening of health disparities (Luck, 2025). These policy contexts may be of particular importance for Latino adults, who experience a greater burden of disability than non-Latino White adults (Garcia et al., 2019; Hayward et al., 2014). Additionally, as we elaborate below, the extensive economic and social policy changes in recent decades—including reduced wages and labor protections for poor and working-class people, the expiration of healthcare subsidies, cuts to social welfare programs, and more restrictive and punitive immigration policies—may have had especially disparate impacts on Latinos.

Although past research emphasizes the role of states in shaping disability for the overall U.S. adult population (Farina et al., 2021; Montez et al., 2017), less is known about the role of more macro-level, broad state policy contexts and ideologies, such as policy liberalism, in shaping disability risks among diverse aging U.S. Latino populations. In this study, we use a political economy approach (Lynch, 2023) and apply insights from Fundamental Cause Theory (FCT; Phelan et al., 2010) to investigate the roles of broad state-level political environments in shaping disability risks among Latino adults in the United States.

FCT emphasizes how structural inequalities affect individuals' health through interactions between macro-level systems and institutions, resulting in unequal allocation of power and resources that manifest in disparate social, economic, and environmental conditions, thereby patterning health risks (Phelan et al., 2010). Although FCT identifies structural systems of inequality as the core drivers of population health patterns, research taking a political economy approach identifies political and policy decisions as the “causes of the causes” of health and health inequalities (Bambra et al., 2019), as they directly and indirectly shape the social and structural determinants of health. In this study, we theorize that state-level economic and social policies may be particularly salient for aging Latino adults, given their disproportionate exposure to hazardous and taxing work conditions, restricted healthcare access, and relatively high levels of economic insecurity, factors that contribute to higher levels of health and disability burden in late life (Padilla & Reyes, 2024; Patti & Boen, 2018; Philbin et al., 2018; Schut & Boen, 2022; Wallace et al., 2019). Thus, from both a political economy and FCT perspective, we argue that state policy contexts are critical drivers of structural inequalities in health and disability, including among Latinos, as they expand or constrain access to health-promoting opportunities and resources. In this sense, disparities in disability risk among aging Latino adults are shaped not only by individual risk and protective factors but also by the broader policy environments in which they live, work, and age.

Importantly, economic and social policy contexts may influence health through a variety of shared and distinct pathways. For example, economic policies may primarily influence critical economic determinants of disability, such as wages, levels of income inequality, and labor market conditions that restrict or expand access to flexible resources, ultimately shaping disparities in disability. In contrast, social policies might target other social determinants of health (e.g., education, access to healthcare, incarceration, and social services) that may moderate the impacts of economic conditions on health and disability risks and shape individuals’ well-being by affecting access to medical care, social welfare support, sense of belonging, and other health-related resources. Although economic and social policy areas may be closely interconnected, we might expect their association with disability to be distinct, given their differing mechanisms, goals, and potential impacts on subgroups.

In recent years, state political environments have diverged dramatically (Montez & Grumbach, 2023). This is especially true when considering state-level policy orientations towards the treatment of immigrants. Decades of stagnation in federal immigration policy, coupled with growing anti-immigrant sentiment in the United States, have resulted in the passage and enactment of bundles of policies that disproportionately target immigrant populations (i.e., undocumented immigrants, legal permanent residents, and naturalized citizens) and their communities (A. Pillai et al., 2025; D. Pillai & Agrita, 2025). These policies have generally restricted immigrants’ access to education, healthcare, and other economic and social services, with significant short- and long-term effects on the health of immigrants, their children, and U.S.-born co-ethnics (Padilla & Reyes, 2024; Philbin et al., 2018; Wallace et al., 2019). Though research identifies immigrant policies as important drivers of health, little is known about how rapidly changing, broad-scale social and political contexts, both nationally and across U.S. states, have impacted the health and well-being of Latino adults. This is because most research in this area focuses narrowly on immigrant-specific policies without considering how broad-scale changes in state policy contexts impact Latino aging.

Thus, this study seeks to address this gap by examining how U.S. states’ policy contexts are associated with disability patterns among mid-life working-age Latino adults, a population at increased risk for disability. Specifically, we build on prior literature by 1) focusing on the Latino population, which comprises a growing share of the older U.S. population that experiences particularly high risks of physical and cognitive disability, 2) examining within-group heterogeneity among Latino subgroups by nativity and citizenship status, and 3) moving beyond specific state policies to focus on how aggregate dimensions of state policy context – namely, economic, social, and overall liberalism – shape disability risk among aging Latino adults. The findings from this study provide new evidence on the role of state policy contexts on disability among the growing and increasingly diverse population of Latino adults in the United States.

## Data and Methods

**Fig. 1:**
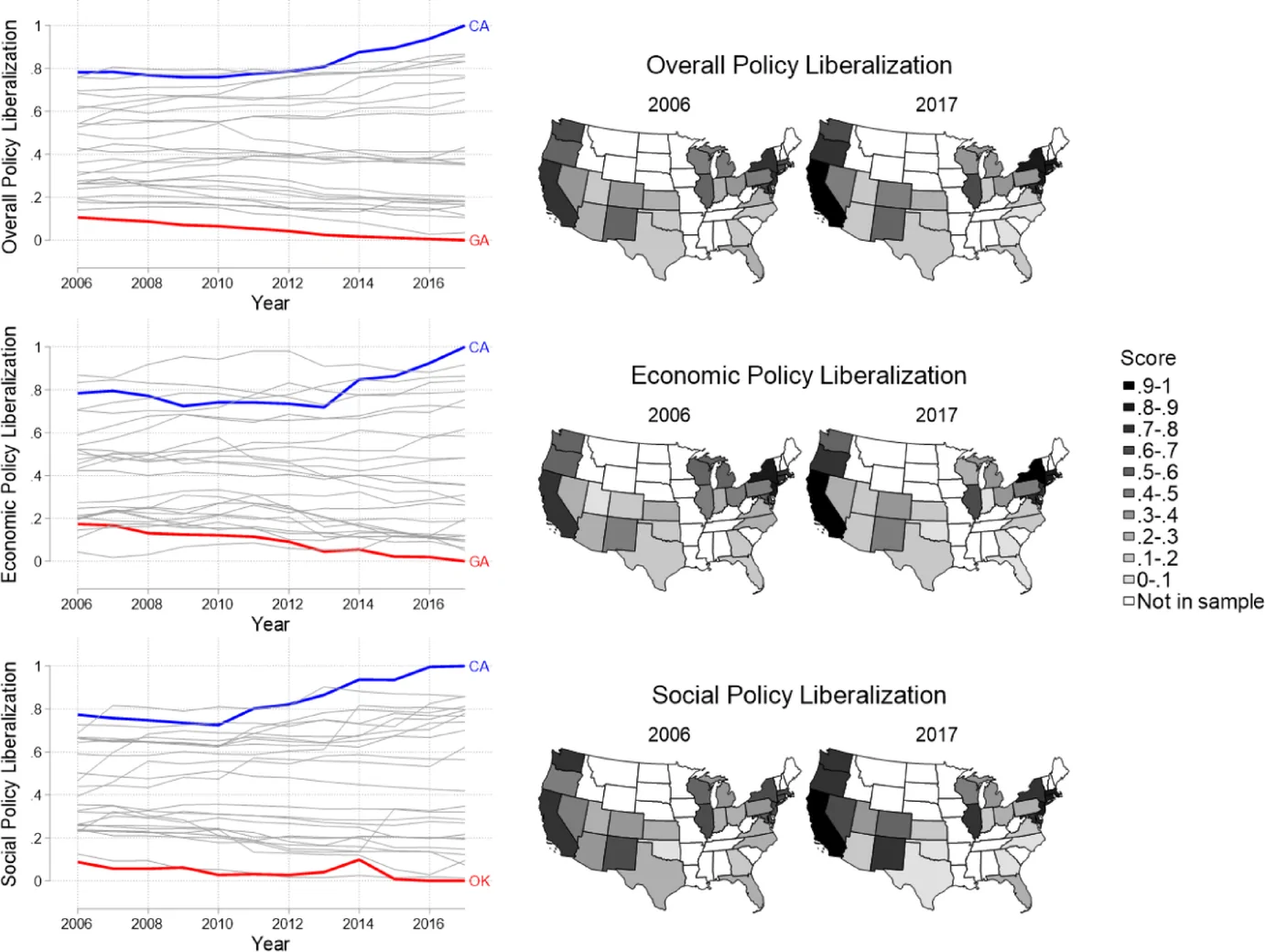
Overall, Economic, and Social Policy Liberalization Scores over Time for the 26 States Represented in the Analytic Sample

This study used the 2008–2019 American Community Survey (ACS) public-use microdata sample, downloaded from IPUMS (Ruggles et al., 2024). The ACS is uniquely suited for this analysis because of its large sample of Latino adults across U.S. states, which enables disaggregation by nativity and citizenship status. We began our study period in 2008, the year the ACS introduced a new set of disability questions, which were not comparable to data from previous years (Erickson et al., 2010). Given state-level variation in policy responses to COVID-19 (Adolph et al., 2021) and trends in COVID-related morbidity and mortality (Luck et al., 2023; Park et al., 2021), as well as concerns regarding data collection during the COVID era (Arias et al., 2025), we focus on trends prior to the pandemic. ACS data was linked to annual state policy data from the State Policy and Politics Database (SPPD) using state FIPS codes. Following prior research, we lagged the policy data by two years (i.e., policy data for year *t* are merged with disability data for year *t + 2*) to ensure the policy was implemented before the disability outcome was measured (Kemp et al., 2022). In supplementary analysis, we examined 1-year and 3-year lags (see Online Supplement Tables S1–S4). Our analytic sample was restricted to Latino adults aged 45–67 years (eligible to participate in the labor force) who reside in one of 26 states that together comprise 95% of the Latino population in this age range (*n* = 1,103,993). The remaining 24 states were excluded because of their small Latino population sizes during the 12-year study period. Economic, social, and overall liberalism scores for the states included in the analytic sample are shown in Fig. 1, revealing a polarizing trend in policy contexts between 2006 and 2017.

### Measures

Our primary outcome was *disability*. We created a dichotomous measure of overall disability, indicating whether an individual reported having any disability (1 = disabled) or reported no disability (0 = not disabled). We also examined four disability outcomes separately: *cognitive difficulty* (e.g., concentrating, remembering, or making decisions), *ambulatory difficulty* (e.g., walking or climbing stairs), *independent living difficulty* (e.g., shopping or visiting a doctor’s office), and *self-care difficulty* (e.g., dressing or bathing) to better document disability patterns among aging Latino adults. Due to word limit constraints, we focus on overall disability in the main body of the paper and present the specific disability measures in the Online Supplement (see Tables S1–S4 and Figures S1–S12).

The three independent variables of interest were *overall*, *economic*, and *social policy liberalism/conservatism*. These measures were developed by Caughey and Warshaw (2016) and updated through 2020 (Caughey & Warshaw, 2024). The measures integrate information on 148 state-level policies across a wide range of policy domains, including civil rights, criminal justice, health behaviors, labor, social welfare, and taxes to create a continuous measure of state liberalism/conservativism, as well as two domains of state liberalism: economic and social. The economic domain encompasses several key policy areas, including social welfare, taxation, labor, and environment. The social domain encompasses policy areas such as women’s rights, moral legislation, family planning, criminal justice, drugs, and religion. Briefly, liberal policies expand the state's power to regulate the economy, protect the rights of historically marginalized groups, and restrict the state's power to punish deviant behavior, whereas conservative policies do the opposite.^1^ The three policy liberalism measures were normalized across the years and states represented in our data, providing a range of actual scores. For instance, a state-year score of 0 indicates that the state in that year had the most conservative configuration of that policy domain across all state-year observations (i.e., all combinations of the 26 states and 12 years represented in our data). We lagged the policy data by 2 years (e.g., policy data for 2006 were merged with disability data in 2008) to ensure the policy was implemented before the disability outcome was measured and to reduce concerns about biases from interstate migration.

The two key moderators were *nativity status* (foreign-born and U.S.-born) and *citizenship status* (citizen, naturalized, and non-citizen). We coded citizens living in U.S. territories as foreign-born, as these individuals do not have the same rights, protections, or access to economic and social programs on the mainland (Hammond, 2021). Latinos born abroad to American citizens were coded as citizens. All models adjust for *age* in years, *sex* (male and female), country of origin or *heritage* (Mexico, Puerto Rico, Cuba, and other countries), *education* (< 12 vs. ≥ 12 years of education), *health insurance* (any vs. none), *employment status* (employed, unemployed, not in the labor force), *marital status* (married vs. otherwise), *number of children*, *metro status* (in metropolitan area vs. not in metropolitan area or mixed), an indicator for having *moved from another state or country* in the last year, and several annual state-level variables including *percent population Latino* (weighted state-level ACS measure), *percent foreign-born* (weighted state-level ACS measure), p*ercent with a bachelor’s degree or higher* (weighted state-level ACS measure of adults aged ≥ 25), *percent unemployment* (University of Kentucky Center for Poverty Research, 2025), and an indicator for *Medicaid expansion* (expanded vs. did not expand; from SPPD). We also included fixed effects for states and years to capture time-invariant state characteristics and time-specific factors not accounted for in the models.

### Analytic Plan

We conducted a series of logit regression models to examine how state policy contexts are associated with disability risk and to assess whether these associations differ by nativity and citizenship status. We first examined each policy liberalism measure in separate models. We next examined: 1) interactions between nativity status and the policy measures in the adjusted model, and 2) interactions between citizenship status and the policy measures in the adjusted model. Following current best practices for interpreting nonlinear interaction effects, we tested average marginal effects (AMEs) and differences in average marginal effects (secondary differences), and present predicted probabilities in the figures below (Mize, 2019). Full tables of odds ratios can be found in Tables S5–S9 of the Online Supplement. All models included standard errors clustered by state and were estimated in Stata 19.

## Results

Table 1 presents key characteristics of our analytic sample of Latino adults aged 45–67 years from 2008 to 2019 residing in one of the 26 states that comprise 95% of the U.S. Latino population. Overall disability prevalence was higher among U.S.-born Latino adults (16.8%) than foreign-born Latino adults (10.9%), and among citizens (17.6%) compared to naturalized citizens (10.0%) and non-citizens (9.3%). The average state liberalism score across all three policy measures was higher for foreign-born Latino adults than for U.S.-born Latino adults (all: 0.53 vs. 0.47; economic: 0.51 vs. 0.45; social: 0.53 vs. 0.49, respectively), and for naturalized citizens than for citizens and non-citizens (all: 0.54 vs. 0.48 and 0.52; economic: 0.52 vs. 0.46 and 0.50; social: 0.55 vs. 0.49 and 0.53, respectively). The average age of the sample was 54 years. Approximately 10% of foreign-born Latino adults were U.S. citizens. The majority of the sample was of Mexican heritage (59.1%), followed by Puerto Rican (9.9%) and Cuban (2.6%) heritage. The remaining 25.4% of the sample, representing various other countries of origin/heritage, were grouped to ensure sufficiently large cell sizes for meaningful analysis.

**Table 1:** Descriptive Statistics for Latino Adults Aged 45–67 Years Residing in Select U.S. States (ACS 2008–2019)

		Pan-ethnic (n = 1,103,993)		USB (n = 421,580)		FB (n = 682,413)		Citizen (n = 489,610)		Naturalize (n = 314,304)		Non-Citizen (n = 300,079)	
	Range	Mean/Prop	S.D	Mean/Prop	S.D	Mean/Prop	S.D	Mean/Prop	S.D	Mean/Prop	S.D	Mean/ Prop	S.D
Difficulty													
Any Difficulties	0,1	0.132		**0.168**		**0.109**		**0.176**		**0.100**		**0.093**	
Cognitive	0,1	0.055		**0.074**		**0.043**		**0.078**		**0.037**		**0.035**	
Ambulatory	0,1	0.099		**0.127**		**0.081**		**0.133**		**0.076**		**0.068**	
Independent Living	0,1	0.056		**0.073**		**0.045**		**0.077**		**0.039**		**0.038**	
Self-Care	0,1	0.035		**0.045**		**0.028**		**0.047**		**0.026**		**0.023**	
Policy Domain													
Overall	0–1	0.510	0.302	**0.476**	**0.398**	**0.531**	**0.303**	**0.481**	**0.295**	**0.542**	**0.304**	**0.524**	**0.307**
Economic	0–1	0.487	0.323	**0.448**	**0.314**	**0.512**	**0.325**	**0.458**	**0.317**	**0.520**	**0.327**	**0.501**	**0.323**
Social	0–1	0.515	0.308	**0.485**	**0.307**	**0.533**	**0.307**	**0.486**	**0.301**	**0.545**	**0.309**	**0.531**	**0.313**
Moderators													
Foreign-born	0,1	0.618						0.139					
Citizenship													
Citizen	0,1	0.444				0.100							
Naturalized	0,1	0.285				0.461							
Non-Citizen	0,1	0.272				0.440							
Covariates													
Female	0,1	0.519		**0.523**		**0.517**		**0.525**		**0.532**		**0.497**	
Age	45–67	54.387	6.392	**54.557**	**6.390**	**54.283**	**6.391**	**54.731**	**6.435**	**55.066**	**6.394**	**53.115**	**6.137**
Heritage													
Mexico	0,1	0.591		**0.686**		**0.532**		**0.605**		**0.499**		**0.664**	
Puerto Rico	0,1	0.099		**0.124**		**0.084**		**0.219**		**0.005**		**0.004**	
Cuba	0,1	0.056		**0.027**		**0.074**		**0.025**		**0.106**		**0.054**	
Other	0,1	0.254		**0.163**		**0.310**		**0.152**		**0.391**		**0.279**	
Health Insurance	0,1	0.773		**0.868**		**0.715**		**0.869**		**0.825**		**0.563**	
High School or Higher	0,1	0.683		0.848		0.581		0.834		0.689		0.431	
Employment													
Employed	0,1	0.641		**0.627**		**0.650**		**0.612**		**0.691**		**0.635**	
Unemployed	0,1	0.045		**0.041**		**0.047**		**0.041**		**0.040**		**0.055**	
Not in labor force	0,1	0.315		**0.332**		**0.304**		**0.348**		**0.269**		**0.309**	
Married	0,1	0.644		**0.590**		**0.678**		**0.584**		**0.709**		**0.675**	
Number of Children	0–9	0.986	1.151	**0.728**	**1.012**	**1.145**	**1.203**	**0.721**	**1.004**	**1.112**	**1.152**	**1.286**	**1.274**
Not in Metro	0,1	0.092		**0.139**		**0.063**		**0.127**		**0.051**		**0.077**	
Moved State/Country	0,1	0.015		**0.012**		**0.017**		**0.014**		**0.009**		**0.022**	
Annual State													
% Latino	2.6–49.3	29.502	11.331	**31.133**	**11.492**	**28.494**	**11.111**	**29.625**	**11.855**	**28.988**	**10.667**	**29.839**	**11.111**
% Foreign-born	3.7–27.3	19.428	6.387	**18.366**	**6.629**	**20.084**	**6.142**	**18.290**	**6.515**	**20.644**	**5.899**	**20.010**	**6.355**
% BA	21.5–45	30.873	3.949	**30.472**	**3.926**	**31.121**	**3.942**	**30.705**	**4.101**	**31.162**	**3.866**	**30.847**	**3.760**
Unemployment	2.3–13.7	6.558	2.468	**6.396**	**2.379**	**6.658**	**2.517**	**6.408**	**2.361**	**6.637**	**2.548**	**6.720**	**2.540**
Medicaid Expansion	0,1	0.230		0.229		0.230		**0.225**		**0.244**		**0.224**	

USB = US-born, FB = foreign-born, S.D. = standard deviation; bold indicates statistically significant (*p* < .05) difference by nativity or citizenship (chi-square and t-tests)

For conciseness, we discuss results from models predicting any disability, which encompass results from models predicting cognitive, ambulatory, independent living, and self-care disability outcomes. Below, we elaborate on three main findings.

**Fig. 2:**
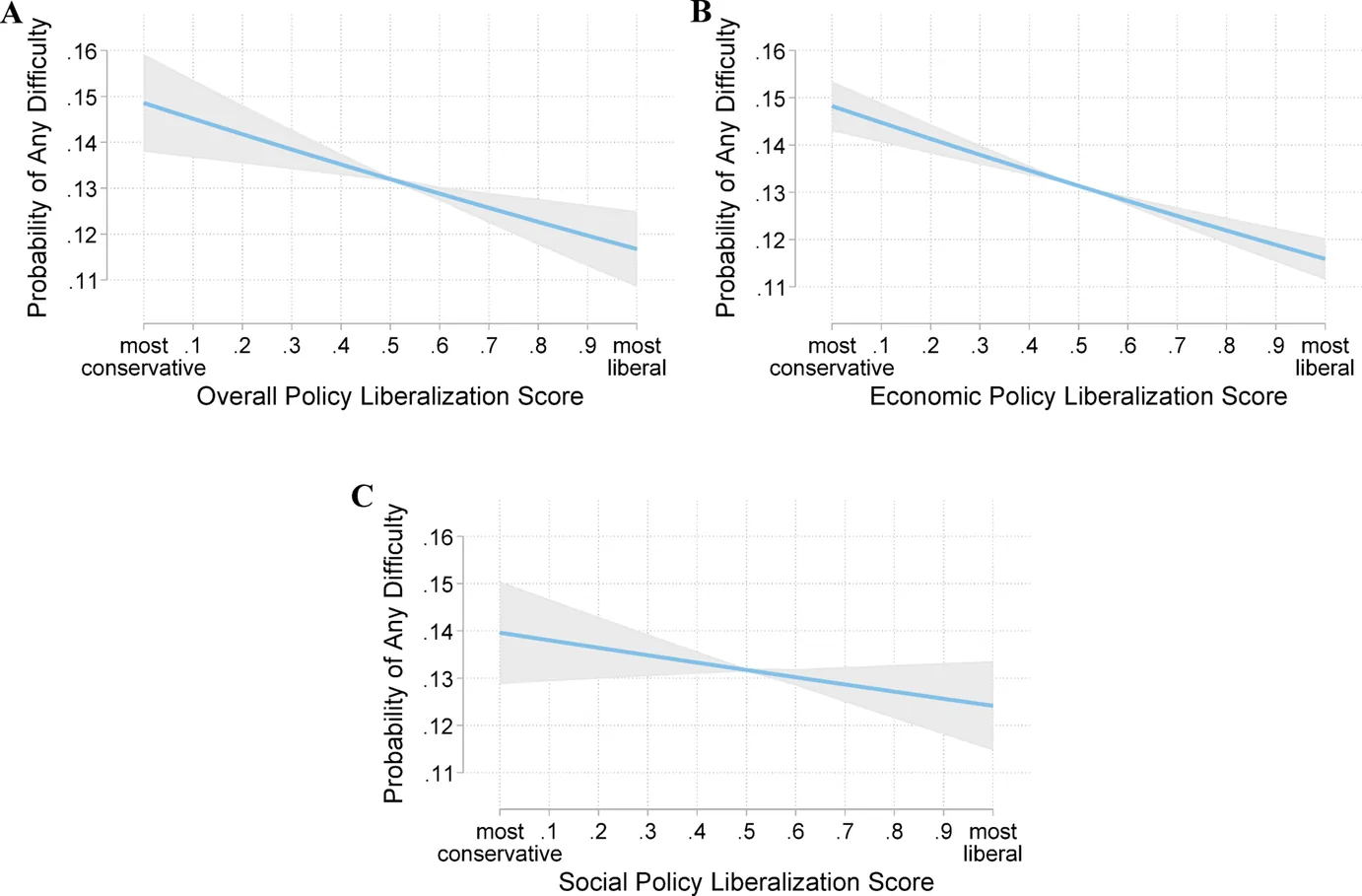
Predicted Probabilities of Any Difficulty among Pan-Ethnic Latino Adults Aged 45–67 Years by Overall (Panel A), Economic (Panel B), and Social (Panel C) Policy Liberalization Note. Predicted probabilities are from adjusted models (corresponding AMEs are in Table 2); the shaded areas represent the 95% confidence interval

First, overall policy liberalism was associated with a lower probability of any disability among Latino adults. This association was potentially driven by economic (rather than social) policy liberalism. Figure 2, Panel A shows that the probability of having any disability was 14.9% in a state with the lowest overall policy liberalism score across all state-year observations, compared to 11.7% in a state with the highest policy liberalism score across state-year observations. The average marginal effect (AME) of overall policy liberalism predicting any disability among Latinos was -0.032 (*p* < .001; Table 2), indicating that each 0.1 overall policy liberalism score increase (moving from conservative to liberal) was associated with a 3-percentage point decrease in the probability of having any disability. When the two domains of the overall policy liberalism score (i.e., economic and social) were examined separately, we found that economic policy liberalism was negatively associated with any disability (AME = -0.032, *p* < .001). Figure 2, Panel B, shows the predicted probabilities associated with economic liberalism. In contrast, the social policy liberalism domain was not associated with reporting any disability, as shown in Fig. 2, Panel C (AME = -0.015, *p* > .05).

**Table 2:** Average Marginal Effects (Standard Errors) and Differences in Average Marginal Effects of Policies for Pan-Ethnic Latino Adults and Latino Subgroups Aged 45–67 Years by Nativity and Citizenship

	Pan-ethnic	USB	FB	Nativity Difference	Citizen	Naturalized	Non-Citizen	Cit. v. Nat. Difference	Cit. v. Non-Cit. Difference	Nat. v. Non-Cit. Difference
Any										
Overall	− 0.032***	− 0.041***	− 0.023*	0.018***	− 0.043***	− 0.021*	− 0.022*	0.022***	0.021***	− 0.001
	(0.010)	(0.009)	(0.011)	(0.003)	(0.010)	(0.010)	(0.009)	(0.002)	(0.003)	(0.002)
Economic	− 0.032***	− 0.042***	− 0.026***	0.016***	− 0.043***	− 0.023***	− 0.023***	0.021***	0.020***	− 0.001
	(0.005)	(0.006)	(0.005)	(0.003)	(0.006)	(0.004)	(0.005)	(0.002)	(0.003)	(0.002)
Social	− 0.015	− 0.024*	− 0.008	0.015***	-0.025*	− 0.006	-0.008	0.019***	0.017***	− 0.002
	(0.010)	(0.010)	(0.011)	(0.003)	(0.011)	(0.011)	(0.010)	(0.002)	(0.003)	(0.002)
Cognitive										
Overall	− 0.028**	− 0.029***	− 0.027**	0.002	− 0.033***	− 0.023**	− 0.022**	0.010***	0.011**	0.001
	(0.009)	(0.009)	(0.009)	(0.003)	(0.010)	(0.009)	(0.007)	(0.003)	(0.004)	(0.002)
Economic	− 0.017***	− 0.018***	− 0.016***	0.002	− 0.021***	− 0.014***	− 0.013***	0.007**	0.008*	0.001
	(0.004)	(0.005)	(0.004)	(0.003)	(0.005)	(0.004)	(0.004)	(0.003)	(0.003)	(0.002)
Social	− 0.016	− 0.016*	− 0.016	0.001	− 0.019*	− 0.013	− 0.014	0.006*	0.005	− 0.001
	(0.009)	(0.008)	(0.009)	(0.003)	(0.010)	(0.009)	(0.007)	(0.003)	(0.003)	(0.002)
Ambulatory										
Overall	− 0.017	− 0.028**	− 0.006	0.022***	− 0.029**	-0.004	-0.005	0.024***	0.024***	− 0.001
	(0.010)	(0.010)	(0.010)	(0.002)	(0.010)	(0.010)	(0.009)	(0.002)	(0.003)	(0.003)
Economic	− 0.024***	− 0.035***	− 0.015**	0.020***	− 0.036***	− 0.012*	− 0.013*	0.024***	0.024***	0.000
	(0.005)	(0.005)	(0.005)	(0.002)	(0.005)	(0.005)	(0.005)	(0.002)	(0.002)	(0.002)
Social	-0.006	− 0.017*	0.003	0.020***	-0.017	0.005	0.003	0.022***	0.020**	− 0.001
	(0.008)	(0.008)	(0.009)	(0.002)	(0.009)	(0.009)	(0.007)	(0.002)	(0.003)	(0.003)
Indep. Living										
Overall	− 0.008	− 0.011	-0.006	0.005*	-0.013	-0.003	-0.007	0.010**	0.006	− 0.004**
	(0.008)	(0.009)	(0.007)	(0.002)	(0.009)	(0.007)	(0.006)	(0.003)	(0.004)	(0.001)
Economic	− 0.012***	− 0.015***	− 0.011***	0.004	− 0.017***	− 0.007*	− 0.010**	0.010***	0.006*	− 0.004***
	(0.004)	(0.004)	(0.003)	(0.002)	(0.004)	(0.003)	(0.003)	(0.002)	(0.002)	(0.001)
Social	− 0.004	− 0.007	− 0.002	0.005*	-0.007	0.001	− 0.003	0.008**	0.004	− 0.004***
	(0.008)	(0.008)	(0.007)	(0.002)	(0.009)	(0.006)	(0.006)	(0.003)	(0.004)	(0.001)
Self-Care										
Overall	-0.001	− 0.004	0.001	0.005**	-0.005	0.005	− 0.001	0.010***	0.005	− 0.005***
	(0.006)	(0.006)	(0.005)	(0.002)	(0.006)	(0.005)	(0.005)	(0.002)	(0.003)	(0.001)
Economic	− 0.008*	− 0.011**	− 0.006	0.004**	− 0.013***	− 0.002	− 0.006	0.010***	0.006**	-0.004***
	(0.003)	(0.004)	(0.003)	(0.002)	(0.003)	(0.004)	(0.004)	(0.001)	(0.002)	(0.001)
Social	0.004	0.001	0.006	0.005***	0.001	0.009	0.003	0.008***	0.003	− 0.006***
	(0.006)	(0.006)	(0.005)	(0.001)	(0.006)	(0.005)	(0.004)	(0.002)	(0.003)	(0.001)

USB = US-born, FB = foreign-born, Cit. = Citizen, Nat. = Naturalized, Indep. = Independent; *p < .05, **p < .01, ***p < .001

**Fig. 3:**
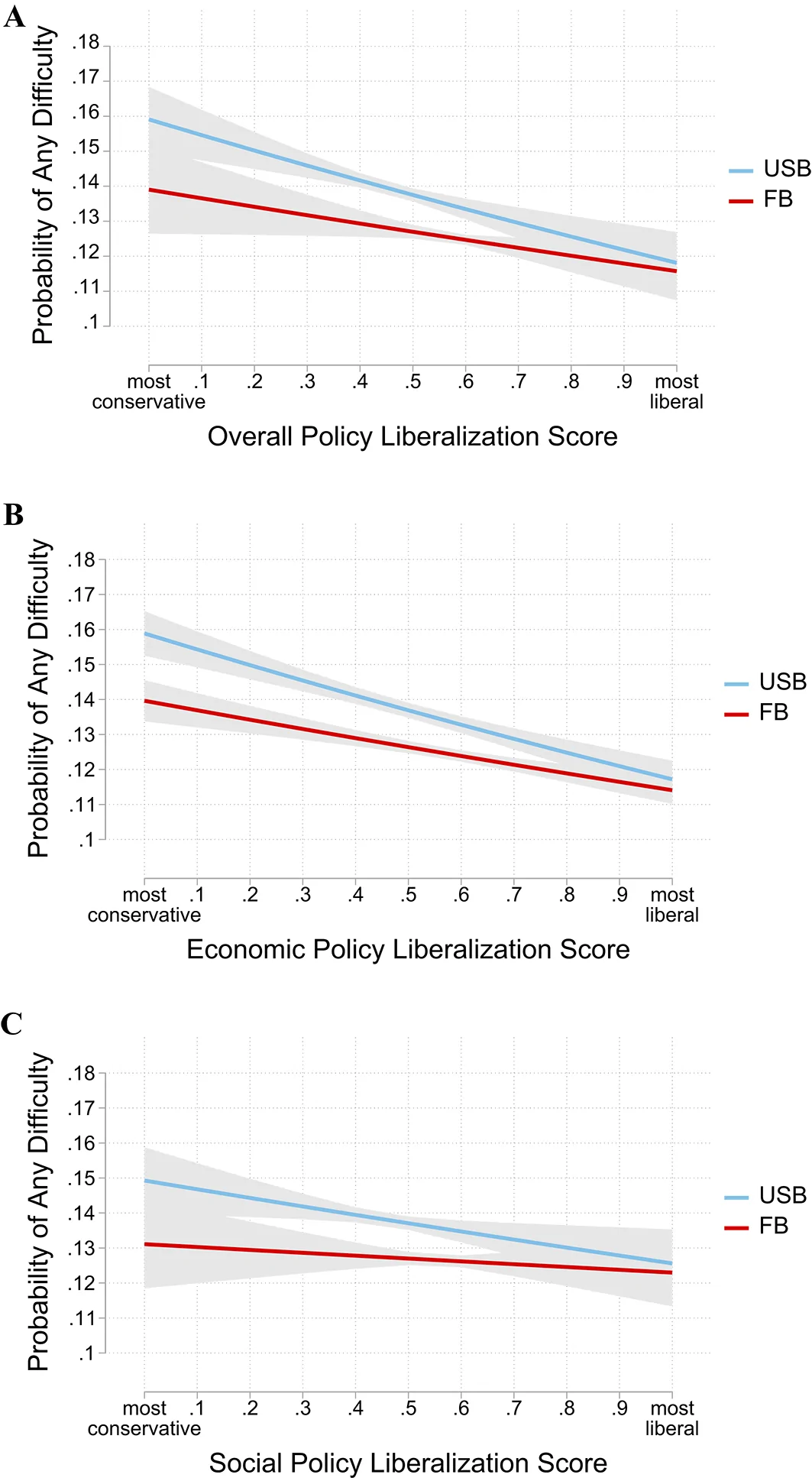
Predicted Probabilities of Any Difficulty among Pan-Ethnic Latino Adults Aged 45–67 Years by Overall (Panel A), Economic (Panel B), and Social (Panel C) Policy Liberalization and Nativity Status Note. Predicted probabilities are from adjusted models (corresponding AMEs are in Table 2); the shaded areas represent the 95% confidence interval

Second, we found strong evidence that more liberal overall, economic, and social state policy environments were associated with reduced disability, particularly for U.S.-born Latino adults aged 45 to 67 years, as seen in Fig. 3, Panels A–C. These findings show that as liberalism scores move from the most conservative to the most liberal, the probability of disability decreases, but the magnitude of the association is not uniform. Liberalism appears to be particularly important for U.S.-born Latino adults, as the AME is statistically larger compared to the AME of foreign-born Latino adults for all three domains of liberalism (see Table 2, “Nativity Difference” column). For example, each 0.1-point increase in economic policy liberalism was associated with a 0.42 percentage point decrease (*p* < .001) in any disability among U.S.-born Latino adults. In contrast, the decrease was 0.26 percentage points (*p* < .01) for foreign-born Latino adults, with a statistically significant difference in AMEs (difference in AMEs = 0.016, *p* < .001). Additionally, whereas overall and economic policy liberalism were negatively associated with disability among U.S.- and foreign-born Latino adults, social policy liberalism was found to be negatively associated with disability among U.S.-born Latino adults only (see Table 2).

**Fig. 4:**
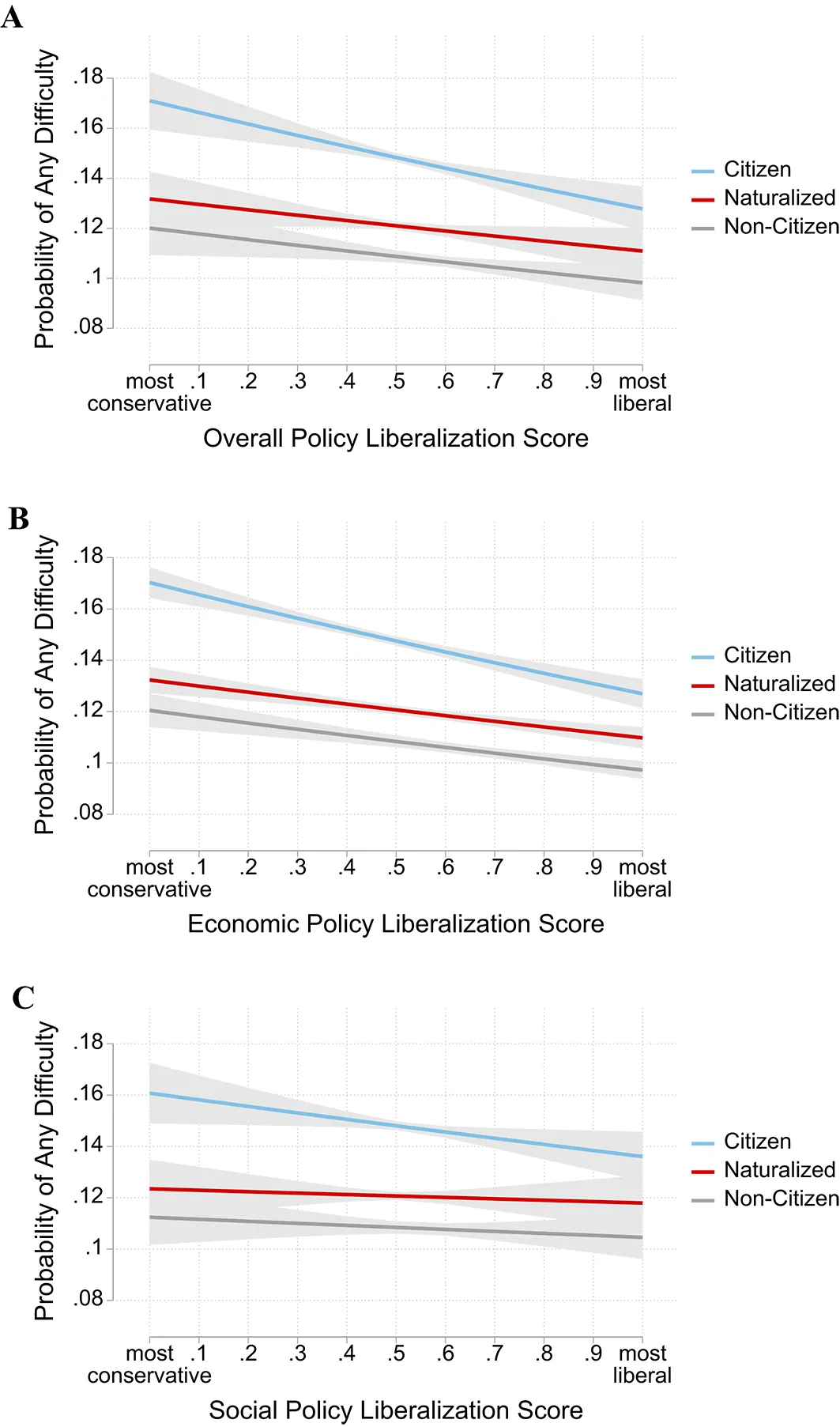
Predicted Probabilities of Any Difficulty among Pan-Ethnic Latino Adults Aged 45–67 Years by Overall (Panel A), Economic (Panel B), and Social (Panel C) Policy Liberalization and Citizenship Status Note. Predicted probabilities are from adjusted models (corresponding AMEs are in Table 2); the shaded areas represent the 95% confidence interval

Third, we found that higher overall and economic policy liberalism scores were associated with reduced disability, particularly among citizens. Figure 4 shows that the probability of having any disability decreased as overall (Panel A) and economic (Panel B) policy liberalism shifts from most conservative to most liberal for all citizenship statuses, with a steeper decline for citizens, as indicated by the significantly larger AMEs for citizens compared to naturalized citizens and non-citizens seen in Table 2. Social policy liberalism was also associated with a lower probability of any disability (Fig. 4, Panel C), but only among citizens (AME = -0.025, *p* < .05). The AME for citizens was significantly different from those for naturalized citizens (difference in AMEs = 0.019; *p* < .001) and non-citizens (difference in AMEs = 0.017, *p* < .001).

To summarize, more liberal policy contexts, particularly more liberal economic policy contexts, were associated with lower probabilities of disability among Latinos. Additionally, the protective effects of liberal policy environments were greater for U.S.-born Latino adults compared to foreign-born Latino adults, and for U.S. Latino citizen adults compared to naturalized and non-citizen Latino adults. In sensitivity analyses, we examined alternative lagging strategies of one (*t + 1*) and three (*t + 3*) years (see Tables S1–S4 in the Online Supplement). The conclusions using these alternative lags were consistent with the results using two-year lags (*t + 2)* shown here.

## Discussion and Conclusion

Prior research examining how state policies shape Latino health and disability outcomes has explicitly focused on immigrant policies. This prior research has documented how anti-immigrant and restrictive immigrant policies create fearful and unwelcoming climates that negatively affect health outcomes, with effects often extending beyond undocumented immigrants to U.S.-born children of immigrants, legal permanent residents, naturalized citizens, and co-ethnics (Padilla & Reyes, 2024; Patti & Boen, 2018; A. Pillai et al., 2025; D. Pillai & Agrita, 2025). The current study contributes to this increasingly relevant area by documenting the role of broader U.S. state policy contexts on disability risk among midlife working-age (45–67 years) Latino adults, with a particular focus on heterogeneity in these links by nativity and citizenship status. State policies that affect people’s ability to avoid economic hardship, protect their rights and dignity, and provide access to health-promoting resources are increasingly understood as critical social determinants of health (Padilla & Reyes, 2024; Philbin et al., 2018; Wallace et al., 2019) and should be considered when assessing the political and policy determinants of Latino health and aging.

Consistent with work taking a political economy approach (Bambra et al., 2019), our results show that broad state political contexts are critical social determinants of Latino disability risks. State contexts structure the economic, social, and physical environments individuals are exposed to, as well as how individuals and communities access resources and navigate systems across the life course (Garcia & García, 2025; Kindratt & Smith, 2024; Montez, 2020). Our results are consistent with the notion that states are institutional actors that shape opportunity (and risk) structures through their social and political climates, affecting Latino subgroups in distinct ways, given variation in how different heritage and immigrant populations are received, supported, and incorporated into local social and economic environments (Vargas et al., 2025).

Whereas prior studies have focused on the roles of specific state-level policies, such as Medicaid expansion, we demonstrate that broader, macro-level political contexts and ideologies, such as policy liberalism, also shape disability outcomes among aging Latino adults. This approach is especially crucial given the increasingly polarized nature of politics in the United States (Montez, 2020; Van Bavel et al., 2024), where these policies often bundle and cluster in ways that research on single policies cannot fully detect (Montez & Grumbach, 2023). Our approach captures the reality that individuals experience multiple policies simultaneously, that states pass bundles of correlated policies, and these policy packages may have synergistic effects that amplify or attenuate health disparities. Indeed, our findings demonstrate the value of examining policy contexts as comprehensive environments rather than isolated policy interventions, highlighting how place-based interventions and policy reforms could be particularly effective in reducing disability disparities among Latino adults. States with more conservative and austere policy contexts may benefit from comprehensive policy reforms that address multiple public policy domains simultaneously, rather than piecemeal approaches that focus on single policy areas.

Our findings also show value in disaggregating the overall policy liberal measure into its two core domains: economic liberalism and social liberalism. Across all models (including all disability outcomes and all lags in the supplementary analysis), we find that economic policy liberalism is consistently associated with lower disability. Given that economic policy influences access to flexible resources, which are versatile in protecting from risks and promoting health, we may expect them to be closely and consistently associated with health and disability (Phelan et al., 2010). The associations were less consistent and somewhat smaller in magnitude for social policy liberalism than for economic policy liberalism. One possible explanation is that the mechanisms linking social policies to disability are less flexible, generally targeting a specific domain (e.g., healthcare, education, criminal justice, etc.). Therefore, the effects of these policies may be less diffuse or direct. Nonetheless, both economic liberalism and social liberalism are associated with reduced disability.

Importantly, we also find that the links between state contexts and disability vary across Latino subgroups. The stark differences in disability risk within the Latino population across states highlight the importance of state contexts for the health of aging Latino adults (Garcia & García, 2025). Our findings further underscore that future research should avoid treating the Latino population as a monolithic group, as this approach obscures important differences in disability risk profiles among Latino subgroups (Garcia et al., 2017, 2020). Notably, we observed differential effects of policy liberalism by nativity and citizenship status. U.S.-born Latino adults experienced greater reductions in disability in more liberal policy contexts compared to foreign-born Latino adults, and citizens experienced greater benefits compared to naturalized citizens and non-citizens. These patterns suggest that the protective effects of liberal policy environments may be most accessible to those with less precarious legal status and greater institutional integration. This finding has important implications for understanding how structural inequalities intersect with policy contexts to create differential health outcomes within Latino communities. The more limited health benefits experienced by foreign-born and non-citizen Latino adults in liberal policy states may reflect barriers to accessing economic and social policy benefits, ongoing vulnerabilities related to legal status, or the insufficient scope of current policies in addressing the needs of these populations. The intersection of state context with heritage and nativity status can affect health outcomes, as different policy environments create unequal exposure to cumulative disadvantages that may manifest in adverse late-life outcomes. Future research should examine different axes of stratification (i.e., education, poverty, and gender) to explore whether this set of policies differently impacts Latino adult populations, who experience longstanding and deeply rooted disparities attributable to social and economic inequalities that directly result from state policies.

Our study is not without limitations. First, our disability measures are based on four ACS functional limitation questions (hearing and vision limitations were excluded). Although the ACS limitation questions are the current standard for measuring disability in U.S. surveys, results using these questions are not generalizable to the disabled population in the U.S., as they do not identify disabled people with functional limitations beyond the measures included, whose disabilities are intermittent, or who do not experience functional limitations (Landes et al., 2025). Second, this study only included state-level policy measures. Policies across municipal and federal levels likely also shape disability risk among midlife Latino adults. Future research should examine how policies interact across multiple levels to create disability disparities among Latino subgroups. Our study is also cross-sectional, which can limit our ability to make inferences. Despite these limitations, this study highlights the importance of considering broad socio-political environments when assessing the disability risks among diverse midlife Latino adults.

## Supplementary Information

Below is the link to the electronic supplementary material.

Supplementary Material 1

## Data Availability

The data that support the findings of this study are publicly available. IPUMS USA data can be accessed at https://usa.ipums.org/usa/. Interested users should create an account and agree to the terms of use when registering to access the data. The State Policy and Politics Database (SPPD) data can be accessed at Open ICPSR 10.3886/E246713V1.

## Footnotes

1 Policy selection was guided by four main criteria: the policy is 1) an output rather than an outcome (e.g., mortality rate) or institution (e.g., government agency), 2) politically salient, 3) comparable across all states (authors converted dollar amounts to be comparable and adjusted for cost of living differences), and 4) available in at least five years (43 policies are available every year). Policies considered economic included social welfare, taxation, environment, and labor, whereas policies considered social included women’s rights, family planning, moral legislation, religion, criminal justice, and drug policy. Policy liberalism and the two disaggregated domains were treated as latent variables, with values inferred from the observed indicators, which were weighted by their ability to discriminate along the latent dimension. Detailed information on the measurement model used to produce the policy liberalism scores is provided in Caughey and Warshaw (2016), where the authors validate their measure.
