# Supplementary material for: Brief Report: State Policy Contexts and Disability Risks Among Midlife Working-Age Latino Adults in the U.S.: Variation by Nativity and Citizenship Status
Source: Popul Res Policy Rev. 2026 Jun 23;45(4):38. doi: 10.1007/s11113-026-10022-6 (PMC13290800; doi:10.1007/s11113-026-10022-6)
Supplement: Supplementary file 1 — Supplementary Material 1 [file 11113_2026_10022_MOESM1_ESM.docx]

# Online Supplement

**Brief Report. State Policy Contexts and Disability Risks Among Midlife Working-Age Latino Adults in the U.S.: Variation by Nativity and Citizenship Status**

| **Table S1.** *Average Marginal Effects (Standard Errors) of Policies (1-, 2-, and 3-year lags) for Pan-Ethnic Latino Adults Aged 45–67 Years* | | | | | |
| --- | --- | --- | --- | --- | --- |
|  | 1-year lag |  | 2-year lag |  | 3-year lag |
| Any Difficulty |  |  |  |  |  |
| Overall | -0.025* |  | -0.025* |  | -0.039*** |
|  | (0.010) |  | (0.011) |  | (0.010) |
| Economic | -0.034*** |  | -0.033*** |  | -0.034*** |
|  | (0.006) |  | (0.008) |  | (0.007) |
| Social | -0.001 |  | -0.007 |  | -0.024** |
|  | (0.010) |  | (0.013) |  | (0.009) |
| Cognitive |  |  |  |  |  |
| Overall | -0.023** |  | -0.023* |  | -0.033*** |
|  | (0.007) |  | (0.009) |  | (0.008) |
| Economic | -0.019*** |  | -0.017*** |  | -0.019** |
|  | (0.003) |  | (0.005) |  | (0.006) |
| Social | -0.010 |  | -0.010 |  | -0.021*** |
|  | (0.008) |  | (0.010) |  | (0.006) |
| Ambulatory |  |  |  |  |  |
| Overall | -0.012 |  | -0.012 |  | -0.022* |
|  | (0.010) |  | (0.010) |  | (0.011) |
| Economic | -0.024*** |  | -0.024*** |  | -0.025*** |
|  | (0.006) |  | (0.006) |  | (0.006) |
| Social | 0.004 |  | -0.001 |  | -0.014 |
|  | (0.008) |  | (0.010) |  | (0.008) |
| Independent Living |  |  |  |  |  |
| Overall | -0.008 |  | -0.004 |  | -0.016* |
|  | (0.006) |  | (0.009) |  | (0.008) |
| Economic | -0.013*** |  | -0.013** |  | -0.017*** |
|  | (0.003) |  | (0.004) |  | (0.005) |
| Social | 0.000 |  | 0.001 |  | -0.012* |
|  | (0.007) |  | (0.009) |  | (0.006) |
| Self-Care |  |  |  |  |  |
| Overall | -0.002 |  | 0.001 |  | -0.005 |
|  | (0.005) |  | (0.006) |  | (0.006) |
| Economic | -0.011** |  | -0.009* |  | -0.008 |
|  | (0.004) |  | (0.004) |  | (0.005) |
| Social | 0.006 |  | 0.007 |  | -0.003 |
|  | (0.006) |  | (0.007) |  | (0.005) |
| **p* < .05, ** *p* < .01, ****p* < .001 | | | | | |

| **Table S2.** *Average Marginal Effects (AMEs) of Policies (1-, 2-, 3-year lag) for Pan-Ethnic Latino Adults Aged 45–67 Years by Nativity* *Status* | | | | | | | | | | | |
| --- | --- | --- | --- | --- | --- | --- | --- | --- | --- | --- | --- |
|  | 1-year lag | | |  | 2-year lag | | |  | 3-year lag | | |
|  | USB | FB | Nativity Difference |  | USB | FB | Nativity Difference |  | USB | FB | Nativity Difference |
| Any Difficulty |  |  |  |  |  |  |  |  |  |  |  |
| All | -0.033*** | -0.017 | 0.017*** |  | -0.036*** | -0.014 | 0.018*** |  | -0.049*** | -0.031** | 0.018*** |
|  | (0.009) | (0.011) | (0.003) |  | (0.010) | (0.012) | -0.003 |  | (0.010) | (0.011) | (0.003) |
| Economic | -0.044*** | -0.028*** | 0.016*** |  | -0.044*** | -0.023** | 0.016*** |  | -0.044*** | -0.027*** | 0.017*** |
|  | (0.006) | (0.006) | (0.003) |  | (0.008) | (0.007) | -0.003 |  | (0.008) | (0.006) | (0.003) |
| Social | -0.009 | 0.006 | 0.014*** |  | -0.018 | 0.004 | 0.015*** |  | -0.033*** | -0.016 | 0.017*** |
|  | (0.010) | (0.011) | (0.003) |  | (0.012) | (0.014) | -0.003 |  | (0.009) | (0.010) | (0.003) |
| Cognitive |  |  |  |  |  |  |  |  |  |  |  |
| All | -0.024*** | -0.022** | 0.002 |  | -0.024** | -0.022* | 0.002 |  | -0.034*** | -0.031*** | 0.003 |
|  | (0.007) | (0.008) | (0.003) |  | (0.008) | (0.010) | -0.003 |  | (0.008) | (0.008) | (0.003) |
| Economic | -0.020*** | -0.019*** | 0.001 |  | -0.019*** | -0.016*** | 0.002 |  | -0.020** | -0.018** | 0.002 |
|  | (0.003) | (0.003) | (0.003) |  | (0.005) | (0.005) | -0.003 |  | (0.007) | (0.006) | (0.003) |
| Social | -0.010 | -0.010 | 0.000 |  | -0.011 | -0.009 | 0.001 |  | -0.022*** | -0.021** | 0.001 |
|  | (0.008) | (0.008) | (0.003) |  | (0.009) | (0.011) | -0.003 |  | (0.006) | (0.006) | (0.003) |
| Ambulatory |  |  |  |  |  |  |  |  |  |  |  |
| All | -0.023* | -0.003 | 0.021*** |  | -0.025* | 0.001 | 0.022*** |  | -0.033** | -0.011 | 0.022*** |
|  | (0.010) | (0.010) | (0.002) |  | (0.010) | (0.011) | -0.002 |  | (0.011) | (0.011) | (0.001) |
| Economic | -0.036*** | -0.016* | 0.020*** |  | -0.037*** | -0.014* | 0.020*** |  | -0.037*** | -0.016* | 0.021*** |
|  | (0.007) | (0.007) | (0.002) |  | (0.006) | (0.007) | -0.002 |  | (0.007) | (0.007) | (0.002) |
| Social | -0.006 | 0.013 | 0.019*** |  | -0.013 | 0.012 | 0.020*** |  | -0.025** | -0.004 | 0.021*** |
|  | (0.008) | (0.008) | (0.002) |  | (0.010) | (0.011) | -0.002 |  | (0.008) | (0.008) | (0.002) |
| Indep. Living |  |  |  |  |  |  |  |  |  |  |  |
| All | -0.011 | -0.006 | 0.005* |  | -0.008 | 0.000 | 0.005* |  | -0.019* | -0.014* | 0.006* |
|  | (0.007) | (0.005) | (0.002) |  | (0.009) | (0.008) | -0.002 |  | (0.009) | (0.007) | (0.002) |
| Economic | -0.015*** | -0.012*** | 0.004* |  | -0.016*** | -0.010* | 0.004 |  | -0.019*** | -0.015*** | 0.004 |
|  | (0.003) | (0.003) | (0.002) |  | (0.005) | (0.004) | -0.002 |  | (0.006) | (0.004) | (0.002) |
| Social | -0.002 | 0.003 | 0.005* |  | -0.002 | 0.006 | 0.005* |  | -0.015* | -0.009 | 0.006** |
|  | (0.008) | (0.006) | (0.002) |  | (0.009) | (0.009) | -0.002 |  | (0.007) | (0.005) | (0.002) |
| Self-Care |  |  |  |  |  |  |  |  |  |  |  |
| All | -0.004 | 0.001 | 0.005*** |  | -0.002 | 0.005 | 0.005** |  | -0.007 | -0.002 | 0.005** |
|  | (0.005) | (0.005) | (0.001) |  | (0.006) | (0.006) | -0.002 |  | (0.006) | (0.005) | (0.002) |
| Economic | -0.014*** | -0.010* | 0.004** |  | -0.011** | -0.006 | 0.004** |  | -0.011 | -0.006 | 0.005* |
|  | (0.004) | (0.004) | (0.001) |  | (0.004) | (0.004) | -0.002 |  | (0.006) | (0.005) | (0.002) |
| Social | 0.003 | 0.009 | 0.005*** |  | 0.003 | 0.011 | 0.005*** |  | -0.006 | 0.000 | 0.006*** |
|  | (0.006) | (0.005) | (0.001) |  | (0.006) | (0.007) | -0.001 |  | (0.005) | (0.004) | (0.001) |
| *Note*. USB=US-born, FB=foreign-born, Cit.=Citizen, Nat.=Naturalized, Indep.=Independent; **p* < .05, ***p* < .01, ****p* < .001 | | | | | | | | | | | |

| **Table S3.** *Average Marginal Effects (AMEs) of Policies (1- and 2-year lag) for Pan-Ethnic Latino Adults Aged 45–67 Years by Citizentship* *Status* |
| --- |

|  | 1-year lag | | | | | | 2-year lag | | | | | |
| --- | --- | --- | --- | --- | --- | --- | --- | --- | --- | --- | --- | --- |
|  | Citizen | Naturalized | Non-Citizen | Cit. v. Nat. Difference | Cit. v. Non-Cit. Difference | Nat. v. Non-Cit. Difference | Citizen | Naturalized | Non-Citizen | Cit. v. Nat. Difference | Cit. v. Non-Cit. Difference | Nat. v. Non-Cit. Difference |
| Any |  |  |  |  |  |  |  |  |  |  |  |  |
| Overall | -0.035*** | -0.015 | -0.016 | 0.023*** | 0.023*** | 0.000 | -0.038** | -0.014 | -0.015 | 0.022*** | 0.021*** | -0.001 |
|  | (0.010) | (0.010) | (0.009) | (0.002) | (0.003) | (0.002) | (0.014) | (0.009) | (0.008) | (0.002) | (0.003) | (0.002) |
| Economic | -0.046*** | -0.025*** | -0.025*** | 0.021*** | 0.021*** | 0.000 | -0.046*** | -0.021*** | -0.023** | 0.021*** | 0.020*** | -0.001 |
|  | (0.006) | (0.005) | (0.006) | (0.002) | (0.003) | (0.002) | (0.009) | (0.006) | (0.007) | (0.002) | (0.003) | (0.002) |
| Social | -0.009 | 0.008 | 0.004 | 0.021*** | 0.020*** | -0.002 | -0.016 | 0.001 | 0.001 | 0.019*** | 0.017*** | -0.002 |
|  | (0.011) | (0.011) | (0.009) | (0.002) | (0.003) | (0.002) | (0.016) | (0.011) | (0.010) | (0.002) | (0.003) | (0.002) |
| Cognitive |  |  |  |  |  |  |  |  |  |  |  |  |
| Overall | -0.027*** | -0.019** | -0.018** | 0.011*** | 0.013*** | 0.002 | -0.030* | -0.017* | -0.018* | 0.010*** | 0.011** | 0.001 |
|  | (0.008) | (0.007) | (0.006) | (0.003) | (0.004) | (0.002) | (0.012) | (0.007) | (0.007) | (0.003) | (0.004) | (0.002) |
| Economic | -0.023*** | -0.016*** | -0.015*** | 0.007** | 0.009** | 0.001 | -0.022** | -0.013*** | -0.013*** | 0.007** | 0.008* | 0.001 |
|  | (0.004) | (0.003) | (0.003) | (0.003) | (0.003) | (0.002) | (0.007) | (0.003) | (0.004) | (0.003) | (0.003) | (0.002) |
| Social | -0.012 | -0.008 | -0.009 | 0.008* | 0.008* | 0.000 | -0.013 | -0.007 | -0.009 | 0.006* | 0.005 | -0.001 |
|  | (0.009) | (0.007) | (0.006) | (0.003) | (0.003) | (0.002) | (0.012) | (0.007) | (0.008) | (0.003) | (0.003) | (0.002) |
| Ambulatory |  |  |  |  |  |  |  |  |  |  |  |  |
| Overall | -0.024* | -0.001 | -0.002 | 0.025*** | 0.025*** | 0.000 | -0.026 | -0.001 | -0.001 | 0.024*** | 0.024*** | -0.001 |
|  | (0.011) | (0.010) | (0.009) | (0.002) | (0.003) | (0.003) | (0.013) | (0.008) | (0.007) | (0.002) | (0.003) | (0.003) |
| Economic | -0.037*** | -0.013* | -0.013* | 0.025*** | 0.024*** | 0.000 | -0.039*** | -0.012* | -0.013* | 0.024*** | 0.024*** | 0.000 |
|  | (0.007) | (0.006) | (0.006) | (0.002) | (0.002) | (0.002) | (0.007) | (0.005) | (0.006) | (0.002) | (0.002) | (0.002) |
| Social | -0.006 | 0.014 | 0.012 | 0.024*** | 0.023*** | -0.001 | -0.0110 | 0.008 | 0.010 | 0.022*** | 0.020** | -0.001 |
|  | (0.009) | (0.009) | (0.007) | (0.002) | (0.003) | (0.002) | (0.013) | (0.008) | (0.007) | (0.002) | (0.003) | (0.003) |
| Indep. Living |  |  |  |  |  |  |  |  |  |  |  |  |
| Overall | -0.012 | -0.003 | -0.007 | 0.011*** | 0.008* | -0.004** | -0.008 | -0.000 | -0.003 | 0.010** | 0.006 | -0.004** |
|  | (0.007) | (0.005) | (0.005) | (0.003) | (0.004) | (0.001) | (0.012) | (0.006) | (0.006) | (0.003) | (0.004) | (0.001) |
| Economic | -0.017*** | -0.008** | -0.011*** | 0.011*** | 0.007* | -0.004** | -0.018** | -0.007* | -0.011** | 0.010*** | 0.006* | -0.004*** |
|  | (0.003) | (0.002) | (0.003) | (0.002) | (0.003) | (0.001) | (0.006) | (0.003) | (0.004) | (0.002) | (0.002) | (0.001) |
| Social | -0.002 | 0.005 | 0.001 | 0.010*** | 0.007* | -0.004** | -0.001 | 0.004 | 0.002 | 0.008** | 0.004 | -0.004*** |
|  | (0.008) | (0.006) | (0.005) | (0.003) | (0.003) | (0.001) | (0.012) | (0.006) | (0.007) | (0.003) | (0.004) | (0.001) |
| Self-Care |  |  |  |  |  |  |  |  |  |  |  |  |
| Overall | -0.006 | 0.004 | -0.001 | 0.011*** | 0.006 | -0.005** | -0.002 | 0.005 | 0.002 | 0.010*** | 0.005 | -0.005*** |
|  | (0.006) | (0.005) | (0.004) | (0.002) | (0.003) | (0.002) | (0.008) | (0.004) | (0.005) | (0.002) | (0.003) | (0.001) |
| Economic | -0.016*** | -0.006 | -0.009* | 0.011*** | 0.006* | -0.004** | -0.014** | -0.003 | -0.007 | 0.010*** | 0.006** | -0.004*** |
|  | (0.004) | (0.004) | (0.004) | (0.002) | (0.003) | (0.001) | (0.004) | (0.003) | (0.004) | (0.001) | (0.002) | (0.001) |
| Social | 0.003 | 0.011* | 0.005 | 0.010*** | 0.005 | -0.005*** | 0.006 | 0.009* | 0.007 | 0.008*** | 0.003 | -0.006*** |
|  | (0.007) | (0.005) | (0.004) | (0.002) | (0.003) | (0.001) | (0.009) | (0.005) | (0.005) | (0.002) | (0.003) | (0.001) |
| *Note*. USB=US-born, FB=foreign-born, Cit.=Citizen, Nat.=Naturalized, Indep.=Independent; **p* < .05, ***p* < .01, ****p* < .001 | | | | | | | | | | | | |

| **Table S4.** *Average Marginal Effects (AMEs) of Policies (3-year lag) for Pan-Ethnic Latino Adults Aged 45–67 Years by Citizentship* *Status* | | | | | | |
| --- | --- | --- | --- | --- | --- | --- |
|  | Citizen | Naturalized | Non-Citizen | Cit. v. Nat. Difference | Cit. v. Non-Cit. Difference | Nat. v. Non-Cit. Difference |
| Any |  |  |  |  |  |  |
| Overall | -0.051*** | -0.028** | -0.028** | 0.023*** | 0.023*** | 0.000 |
|  | (0.011) | (0.010) | (0.009) | (0.002) | (0.003) | (0.002) |
| Economic | -0.046*** | -0.024*** | -0.024*** | 0.021*** | 0.021*** | 0.000 |
|  | (0.008) | (0.006) | (0.006) | (0.002) | (0.003) | (0.002) |
| Social | -0.034*** | -0.013 | -0.015 | 0.021*** | 0.020*** | -0.002 |
|  | (0.009) | (0.010) | (0.008) | (0.002) | (0.003) | (0.002) |
| Cognitive |  |  |  |  |  |  |
| Overall | -0.039*** | -0.028*** | -0.026*** | 0.011*** | 0.013*** | 0.002 |
|  | (0.009) | (0.007) | (0.007) | (0.003) | (0.004) | (0.002) |
| Economic | -0.023** | -0.015** | -0.014** | 0.007** | 0.009** | 0.001 |
|  | (0.007) | (0.005) | (0.005) | (0.003) | (0.003) | (0.002) |
| Social | -0.025*** | -0.017** | -0.018** | 0.008* | 0.008* | 0.000 |
|  | (0.007) | (0.006) | (0.006) | (0.003) | (0.003) | (0.002) |
| Ambulatory |  |  |  |  |  |  |
| Overall | -0.034** | -0.009 | -0.009 | 0.025*** | 0.025*** | 0.000 |
|  | (0.012) | (0.011) | (0.010) | (0.002) | (0.003) | (0.003) |
| Economic | -0.037*** | -0.013* | -0.013* | 0.025*** | 0.024*** | 0.000 |
|  | (0.007) | (0.006) | (0.006) | (0.002) | (0.002) | (0.002) |
| Social | -0.026** | -0.002 | -0.002 | 0.024*** | 0.023*** | -0.001 |
|  | (0.009) | (0.008) | (0.007) | (0.002) | (0.003) | (0.002) |
| Indep. Living |  |  |  |  |  |  |
| Overall | -0.021* | -0.010 | -0.014* | 0.011*** | 0.008* | -0.004** |
|  | (0.009) | (0.007) | (0.006) | (0.003) | (0.004) | (0.001) |
| Economic | -0.021*** | -0.010* | -0.014*** | 0.011*** | 0.007* | -0.004** |
|  | (0.006) | (0.004) | (0.004) | (0.002) | (0.003) | (0.001) |
| Social | -0.016* | -0.006 | -0.010* | 0.010*** | 0.007* | -0.004** |
|  | (0.007) | (0.005) | (0.004) | (0.003) | (0.003) | (0.001) |
| Self-Care |  |  |  |  |  |  |
| Overall | -0.009 | 0.002 | -0.003 | 0.011*** | 0.006 | -0.005** |
|  | (0.007) | (0.006) | (0.005) | (0.002) | (0.003) | (0.002) |
| Economic | -0.013* | -0.002 | -0.006 | 0.011*** | 0.006* | -0.004** |
|  | (0.006) | (0.005) | (0.005) | (0.002) | (0.003) | (0.001) |
| Social | -0.007 | 0.004 | -0.002 | 0.010*** | 0.005 | -0.005*** |
|  | (0.005) | (0.004) | (0.004) | (0.002) | (0.003) | (0.001) |
| *Note*. USB=US-born, FB=foreign-born, Cit.=Citizen, Nat.=Naturalized, Indep.=Independent; **p* < .05, ***p* < .01, ****p* <.001 | | | | | | |

**Figure S1.** *Predicted Probabilities of Cognitive Difficulty among Pan-Ethnic Latino Adults Aged 45–67 Years by Overall (Panel A), Economic (Panel B), and Social (Panel C) Policy Liberalization*

**A**

**B**

**C**

*Note*. Predicted probabilities are from adjusted models (corresponding AMEs are in Table S1, 2-year lag); the shaded areas represent the 95% confidence intervals.

**Figure S2.** *Predicted Probabilities of Ambulatory Difficulty among Pan-Ethnic Latino Adults Aged 45–67 Years by Overall (Panel A), Economic (Panel B), and Social (Panel C) Policy Liberalization*

**C**

**B**

**A**

*Note*. Predicted probabilities are from adjusted models (corresponding AMEs are in Table S1, 2-year lag); the shaded areas represent the 95% confidence intervals.

**Figure S3.** *Predicted Probabilities of Independent Living Difficulty among Pan-Ethnic Latino Adults Aged 45–67 Years by Overall (Panel A), Economic (Panel B), and Social (Panel C) Policy Liberalization*

**A**

**B**

**C**

*Note*. Predicted probabilities are from adjusted models (corresponding AMEs are in Table S1, 2-year lag); the shaded areas represent the 95% confidence intervals.

**Figure S4.** *Predicted Probabilities of Self-Care Difficulty among Pan-Ethnic Latino Adults Aged 45–67 Years by Overall (Panel A), Economic (Panel B), and Social (Panel C) Policy Liberalization*

**B**

**A**

**C**

*Note*. Predicted probabilities are from adjusted models (corresponding AMEs are in Table S1, 2-year lag); the shaded areas represent the 95% confidence intervals.

**Figure S5.** *Predicted Probabilities of Cognitive Difficulty among Pan-Ethnic Latino Adults Aged 45–67 Years by Overall (Panel A), Economic (Panel B), and Social (Panel C) Policy Liberalization and Nativity Status*

**A**

**B**

**C**

*Note*. Predicted probabilities are from adjusted models (corresponding AMEs are in Table S2, 2-year lag); the shaded areas represent the 95% confidence intervals.

**Figure S6.** *Predicted Probabilities of Ambulatory Difficulty among Pan-Ethnic Latino Adults Aged 45–67 Years by Overall (Panel A), Economic (Panel B), and Social (Panel C) Policy Liberalization and Nativity Status*

**B**

**A**

**C**

*Note*. Predicted probabilities are from adjusted models (corresponding AMEs are in Table S2, 2-year lag); the shaded areas represent the 95% confidence intervals.

**Figure S7.** *Predicted Probabilities of Independent Living Difficulty among Pan-Ethnic Latino Adults Aged 45–67 Years by Overall (Panel A), Economic (Panel B), and Social (Panel C) Policy Liberalization and Nativity Status*

**A**

**B**

**CC**

*Note*. Predicted probabilities are from adjusted models (corresponding AMEs are in Table S2, 2-year lag); the shaded areas represent the 95% confidence intervals.

**Figure S8.** *Predicted Probabilities of Self-Care Difficulty among Pan-Ethnic Latino Adults Aged 45–67 Years by Overall (Panel A), Economic (Panel B), and Social (Panel C) Policy Liberalization and Nativity Status*

**C**

**B**

**A**

*Note*. Predicted probabilities are from adjusted models (corresponding AMEs are in Table S2, 2-year lag); the shaded areas represent the 95% confidence intervals.

**Figure S9.** *Predicted Probabilities of Cognitive Difficulty among Pan-Ethnic Latino Adults Aged 45–67 Years by Overall (Panel A), Economic (Panel B), and Social (Panel C) Policy Liberalization and Citizenship Status.*

**A**

**B**

**C**

*Note*. Predicted probabilities are from adjusted models (corresponding AMEs are in Table S3, 2-year lag); the shaded areas represent the 95% confidence intervals.

**Figure S10.** *Predicted Probabilities of Ambulatory Difficulty among Pan-Ethnic Latino Adults Aged 45–67 Years by Overall (Panel A), Economic (Panel B), and Social (Panel C) Policy Liberalization and Citizenship Status*

**A**

**B**

**C**

*Note*. Predicted probabilities are from adjusted models (corresponding AMEs are in Table S3, 2-year lag); the shaded areas represent the 95% confidence intervals.

**Figure S11.** *Predicted Probabilities of Independent Living Difficulty among Pan-Ethnic Latino Adults Aged 45–67 Years by Overall (Panel A), Economic (Panel B), and Social (Panel C) Policy Liberalization and Citizenship Status*

**A**

**B**

**C**

*Note*. Predicted probabilities are from adjusted models (corresponding AMEs are in Table S3, 2-year lag); the shaded areas represent the 95% confidence interval.

**Figure S12.** *Predicted Probabilities of Self-Care Difficulty among Pan-Ethnic Latino Adults Aged 45–67 Years by Overall (Panel A), Economic (Panel B), and Social (Panel C) Policy Liberalization and Citizenship Status*

**A**

**B**

**C**

*Note*. Predicted probabilities are from adjusted models (corresponding AMEs are in Table S3, 2-year lag); the shaded areas represent the 95% confidence intervals.

**Table S5.** *Odds Ratios (Standard Errors) from Fixed Effects Models of Associations Between Policy Liberalization Scores and Any Difficulty (2-year lag) among Pan-Ethnic Latino Adults Aged 45–67 Years, by Nativity and Citizenship*

|  | Model 1 | Model 2 | Model 3 | Model 4 | Model 5 | Model 6 | Model 7 | Model 8 | Model 9 |
| --- | --- | --- | --- | --- | --- | --- | --- | --- | --- |
| Policy |  |  |  |  |  |  |  |  |  |
| Overall | 0.713^***^ |  |  | 0.656^***^ |  |  | 0.658^***^ |  |  |
|  | (0.073) |  |  | (0.062) |  |  | (0.066) |  |  |
| Economic |  | 0.708^***^ |  |  | 0.650^***^ |  |  | 0.656^***^ |  |
|  |  | (0.037) |  |  | (0.037) |  |  | (0.037) |  |
| Social |  |  | 0.848 |  |  | 0.783^*^ |  |  | 0.788^*^ |
|  |  |  | (0.093) |  |  | (0.078) |  |  | (0.082) |
| FB | 0.900^***^ | 0.900^***^ | 0.900^***^ | 0.822^***^ | 0.829^***^ | 0.831^***^ | 0.899^***^ | 0.900^***^ | 0.898^***^ |
|  | (0.020) | (0.020) | (0.020) | (0.025) | (0.026) | (0.026) | (0.019) | (0.019) | (0.019) |
| FB × Policy |  |  |  |  |  |  |  |  |  |
| FB × Overall |  |  |  | 1.183^***^ |  |  |  |  |  |
|  |  |  |  | (0.038) |  |  |  |  |  |
| FB × Economic |  |  |  |  | 1.163^***^ |  |  |  |  |
|  |  |  |  |  | (0.035) |  |  |  |  |
| FB × Social |  |  |  |  |  | 1.168^***^ |  |  |  |
|  |  |  |  |  |  | (0.043) |  |  |  |
| Citizenship (ref.=Citizen) |  |  |  |  |  |  |  |  |  |
| Naturalized | 0.754^***^ | 0.754^***^ | 0.754^***^ | 0.758^***^ | 0.761^***^ | 0.754^***^ | 0.687^***^ | 0.695^***^ | 0.689^***^ |
|  | (0.018) | (0.018) | (0.018) | (0.011) | (0.012) | (0.012) | (0.014) | (0.014) | (0.015) |
| Non-Citizen | 0.652^***^ | 0.651^***^ | 0.652^***^ | 0.656^***^ | 0.659^***^ | 0.652^***^ | 0.605^***^ | 0.611^***^ | 0.607^***^ |
|  | (0.021) | (0.021) | (0.021) | (0.014) | (0.014) | (0.015) | (0.018) | (0.018) | (0.019) |
| Citizenship × Policy |  |  |  |  |  |  |  |  |  |
| Naturalized × Overall |  |  |  |  |  |  | 1.203^***^ |  |  |
|  |  |  |  |  |  |  | (0.028) |  |  |
| Non-Citizen × Overall |  |  |  |  |  |  | 1.166^***^ |  |  |
|  |  |  |  |  |  |  | (0.031) |  |  |
| Naturalized × Economic |  |  |  |  |  |  |  | 1.183^***^ |  |
|  |  |  |  |  |  |  |  | (0.023) |  |
| Non-Citizen × Economic |  |  |  |  |  |  |  | 1.148^***^ |  |
|  |  |  |  |  |  |  |  | (0.027) |  |
| Naturalized × Social |  |  |  |  |  |  |  |  | 1.193^***^ |
|  |  |  |  |  |  |  |  |  | (0.034) |
| Non-Citizen × Social |  |  |  |  |  |  |  |  | 1.155^***^ |
|  |  |  |  |  |  |  |  |  | (0.035) |
| Country of Origin (ref.=Mexico) |  |  |  |  |  |  |  |  |  |
| Puerto Rico | 1.311^***^ | 1.311^***^ | 1.311^***^ | 1.320^***^ | 1.325^***^ | 1.315^***^ | 1.317^***^ | 1.321^***^ | 1.312^***^ |
|  | (0.047) | (0.047) | (0.047) | (0.034) | (0.033) | (0.036) | (0.034) | (0.033) | (0.036) |
| Cuba | 1.191^***^ | 1.192^***^ | 1.191^***^ | 1.198^***^ | 1.199^***^ | 1.197^***^ | 1.205^***^ | 1.207^***^ | 1.204^***^ |
|  | (0.035) | (0.035) | (0.035) | (0.035) | (0.034) | (0.035) | (0.037) | (0.037) | (0.037) |
| Other | 1.048^*^ | 1.048^*^ | 1.048^*^ | 1.047^*^ | 1.046^*^ | 1.048^*^ | 1.045^*^ | 1.044^*^ | 1.047^*^ |
|  | (0.021) | (0.021) | (0.021) | (0.020) | (0.020) | (0.020) | (0.020) | (0.020) | (0.020) |
| Age | 1.011^***^ | 1.011^***^ | 1.011^***^ | 1.011^***^ | 1.011^***^ | 1.011^***^ | 1.011^***^ | 1.011^***^ | 1.011^***^ |
|  | (0.002) | (0.002) | (0.002) | (0.002) | (0.002) | (0.002) | (0.002) | (0.002) | (0.002) |
| Female | 0.836^***^ | 0.836^***^ | 0.836^***^ | 0.836^***^ | 0.836^***^ | 0.836^***^ | 0.836^***^ | 0.836^***^ | 0.836^***^ |
|  | (0.026) | (0.026) | (0.026) | (0.026) | (0.026) | (0.026) | (0.026) | (0.026) | (0.026) |
| Health Insurance | 1.981^***^ | 1.981^***^ | 1.981^***^ | 1.978^***^ | 1.978^***^ | 1.978^***^ | 1.977^***^ | 1.978^***^ | 1.978^***^ |
|  | (0.035) | (0.034) | (0.035) | (0.034) | (0.034) | (0.034) | (0.034) | (0.034) | (0.034) |
| High School | 0.629^***^ | 0.629^***^ | 0.628^***^ | 0.630^***^ | 0.630^***^ | 0.630^***^ | 0.630^***^ | 0.629^***^ | 0.630^***^ |
|  | (0.014) | (0.014) | (0.014) | (0.014) | (0.014) | (0.014) | (0.014) | (0.014) | (0.014) |
| Employment (ref.=Employed) |  |  |  |  |  |  |  |  |  |
| Unemployed | 2.881^***^ | 2.881^***^ | 2.881^***^ | 2.882^***^ | 2.882^***^ | 2.881^***^ | 2.881^***^ | 2.882^***^ | 2.881^***^ |
|  | (0.110) | (0.110) | (0.110) | (0.111) | (0.111) | (0.111) | (0.110) | (0.111) | (0.110) |
| Not in Labor Force | 8.100^***^ | 8.100^***^ | 8.099^***^ | 8.103^***^ | 8.103^***^ | 8.103^***^ | 8.104^***^ | 8.104^***^ | 8.104^***^ |
|  | (0.203) | (0.203) | (0.203) | (0.203) | (0.203) | (0.203) | (0.203) | (0.203) | (0.203) |
| Married | 0.504^***^ | 0.504^***^ | 0.504^***^ | 0.504^***^ | 0.504^***^ | 0.504^***^ | 0.504^***^ | 0.504^***^ | 0.504^***^ |
|  | (0.005) | (0.005) | (0.005) | (0.005) | (0.005) | (0.005) | (0.005) | (0.005) | (0.005) |
| Number of Children | 0.903^***^ | 0.903^***^ | 0.903^***^ | 0.903^***^ | 0.903^***^ | 0.903^***^ | 0.903^***^ | 0.903^***^ | 0.903^***^ |
|  | (0.005) | (0.005) | (0.005) | (0.005) | (0.005) | (0.005) | (0.005) | (0.005) | (0.005) |
| Not in Metro | 1.103^***^ | 1.103^***^ | 1.103^***^ | 1.100^***^ | 1.101^***^ | 1.100^***^ | 1.101^***^ | 1.101^***^ | 1.100^***^ |
|  | (0.028) | (0.028) | (0.028) | (0.028) | (0.028) | (0.028) | (0.028) | (0.028) | (0.028) |
| Moved in Past Year | 0.865^**^ | 0.864^**^ | 0.864^**^ | 0.866^**^ | 0.866^**^ | 0.866^**^ | 0.864^**^ | 0.864^**^ | 0.865^**^ |
|  | (0.041) | (0.041) | (0.041) | (0.041) | (0.041) | (0.041) | (0.041) | (0.041) | (0.041) |
| State-Level |  |  |  |  |  |  |  |  |  |
| % Latino | 0.960^*^ | 0.958^**^ | 0.958^*^ | 0.961^*^ | 0.959^**^ | 0.959^*^ | 0.961^*^ | 0.959^**^ | 0.959^*^ |
|  | (0.015) | (0.015) | (0.017) | (0.015) | (0.015) | (0.017) | (0.016) | (0.015) | (0.017) |
| % Immigrant | 1.064^***^ | 1.058^***^ | 1.071^***^ | 1.063^***^ | 1.058^***^ | 1.070^***^ | 1.064^***^ | 1.058^***^ | 1.071^***^ |
|  | (0.013) | (0.012) | (0.014) | (0.013) | (0.012) | (0.014) | (0.013) | (0.012) | (0.014) |
| Expanded Medicaid | 1.101^***^ | 1.095^***^ | 1.083^**^ | 1.102^***^ | 1.097^***^ | 1.084^**^ | 1.102^***^ | 1.095^***^ | 1.083^**^ |
|  | (0.024) | (0.026) | (0.027) | (0.025) | (0.027) | (0.027) | (0.025) | (0.026) | (0.027) |
| Unemployment Rate | 1.000 | 1.001 | 1.000 | 1.000 | 1.002 | 1.000 | 1.000 | 1.002 | 1.000 |
|  | (0.010) | (0.010) | (0.009) | (0.010) | (0.010) | (0.009) | (0.010) | (0.010) | (0.009) |
| % with BA | 0.977 | 0.979 | 0.972 | 0.977 | 0.980 | 0.973 | 0.977 | 0.980 | 0.973 |
|  | (0.020) | (0.019) | (0.021) | (0.020) | (0.019) | (0.021) | (0.020) | (0.020) | (0.021) |
| Constant | 0.149^**^ | 0.156^**^ | 0.159^*^ | 0.149^**^ | 0.152^**^ | 0.160^*^ | 0.149^**^ | 0.154^**^ | 0.159^*^ |
|  | (0.100) | (0.103) | (0.116) | (0.100) | (0.099) | (0.118) | (0.100) | (0.101) | (0.117) |
| Pseudo *R*^2^ | 0.199 | 0.199 | 0.199 | 0.199 | 0.199 | 0.199 | 0.199 | 0.199 | 0.199 |

*Note*. FB=foreign-born; models adjust for state and year fixed effects and cluster standard errors; ^*^*p* < .05, ^**^*p* < .01, ^***^*p* < .001

**Table S6.** *Odds Ratios (Standard Errors) from Fixed Effects Models of Associations Between Policy Liberalization Scores and Cognitive Difficulty (2-year lag) among Pan-Ethnic Latino Adults Aged 45–67 Years, by Nativity and Citizenship*

|  | Model 1 | Model 2 | Model 3 | Model 4 | Model 5 | Model 6 | Model 7 | Model 8 | Model 9 |
| --- | --- | --- | --- | --- | --- | --- | --- | --- | --- |
| Policy |  |  |  |  |  |  |  |  |  |
| Overall | 0.550^**^ |  |  | 0.547^***^ |  |  | 0.542^***^ |  |  |
|  | (0.104) |  |  | (0.100) |  |  | (0.099) |  |  |
| Economic |  | 0.687^***^ |  |  | 0.682^***^ |  |  | 0.676^***^ |  |
|  |  | (0.063) |  |  | (0.070) |  |  | (0.065) |  |
| Social |  |  | 0.705 |  |  | 0.708 |  |  | 0.702^*^ |
|  |  |  | (0.132) |  |  | (0.125) |  |  | (0.125) |
| FB | 0.916^***^ | 0.917^***^ | 0.916^***^ | 0.912^*^ | 0.909^*^ | 0.921 | 0.916^***^ | 0.917^***^ | 0.916^***^ |
|  | (0.017) | (0.017) | (0.017) | (0.041) | (0.039) | (0.042) | (0.016) | (0.016) | (0.017) |
| FB × Policy |  |  |  |  |  |  |  |  |  |
| FB × Overall |  |  |  | 1.009 |  |  |  |  |  |
|  |  |  |  | (0.063) |  |  |  |  |  |
| FB × Economic |  |  |  |  | 1.014 |  |  |  |  |
|  |  |  |  |  | (0.059) |  |  |  |  |
| FB × Social |  |  |  |  |  | 0.991 |  |  |  |
|  |  |  |  |  |  | (0.066) |  |  |  |
| Citizenship (ref.=Citizen) |  |  |  |  |  |  |  |  |  |
| Naturalized | 0.694^***^ | 0.693^***^ | 0.694^***^ | 0.694^***^ | 0.694^***^ | 0.694^***^ | 0.676^***^ | 0.677^***^ | 0.679^***^ |
|  | (0.015) | (0.016) | (0.015) | (0.014) | (0.013) | (0.016) | (0.026) | (0.023) | (0.028) |
| Non-Citizen | 0.582^***^ | 0.582^***^ | 0.582^***^ | 0.582^***^ | 0.582^***^ | 0.582^***^ | 0.581^***^ | 0.576^***^ | 0.591^***^ |
|  | (0.011) | (0.011) | (0.011) | (0.011) | (0.010) | (0.011) | (0.026) | (0.025) | (0.026) |
| Citizenship × Policy |  |  |  |  |  |  |  |  |  |
| Naturalized × Overall |  |  |  |  |  |  | 1.051 |  |  |
|  |  |  |  |  |  |  | (0.054) |  |  |
| Non-Citizen × Overall |  |  |  |  |  |  | 1.004 |  |  |
|  |  |  |  |  |  |  | (0.065) |  |  |
| Naturalized × Economic |  |  |  |  |  |  |  | 1.050 |  |
|  |  |  |  |  |  |  |  | (0.046) |  |
| Non-Citizen × Economic |  |  |  |  |  |  |  | 1.022 |  |
|  |  |  |  |  |  |  |  | (0.064) |  |
| Naturalized × Social |  |  |  |  |  |  |  |  | 1.041 |
|  |  |  |  |  |  |  |  |  | (0.058) |
| Non-Citizen × Social |  |  |  |  |  |  |  |  | 0.972 |
|  |  |  |  |  |  |  |  |  | (0.062) |
| Country of Origin (ref.=Mexico) |  |  |  |  |  |  |  |  |  |
| Puerto Rico | 1.302^***^ | 1.302^***^ | 1.302^***^ | 1.302^***^ | 1.303^***^ | 1.302^***^ | 1.302^***^ | 1.304^***^ | 1.301^***^ |
|  | (0.046) | (0.046) | (0.046) | (0.045) | (0.043) | (0.046) | (0.045) | (0.043) | (0.046) |
| Cuba | 1.553^***^ | 1.554^***^ | 1.553^***^ | 1.554^***^ | 1.555^***^ | 1.553^***^ | 1.557^***^ | 1.559^***^ | 1.555^***^ |
|  | (0.064) | (0.063) | (0.064) | (0.061) | (0.061) | (0.061) | (0.061) | (0.061) | (0.061) |
| Other | 1.160^***^ | 1.160^***^ | 1.160^***^ | 1.160^***^ | 1.160^***^ | 1.160^***^ | 1.160^***^ | 1.159^***^ | 1.160^***^ |
|  | (0.027) | (0.027) | (0.027) | (0.027) | (0.027) | (0.027) | (0.027) | (0.026) | (0.027) |
| Age | 0.976^***^ | 0.976^***^ | 0.976^***^ | 0.976^***^ | 0.976^***^ | 0.976^***^ | 0.976^***^ | 0.976^***^ | 0.976^***^ |
|  | (0.002) | (0.002) | (0.002) | (0.002) | (0.002) | (0.002) | (0.002) | (0.002) | (0.002) |
| Female | 0.730^***^ | 0.731^***^ | 0.730^***^ | 0.730^***^ | 0.730^***^ | 0.730^***^ | 0.730^***^ | 0.730^***^ | 0.731^***^ |
|  | (0.022) | (0.022) | (0.022) | (0.022) | (0.022) | (0.022) | (0.022) | (0.022) | (0.022) |
| Health Insurance | 2.050^***^ | 2.049^***^ | 2.050^***^ | 2.050^***^ | 2.049^***^ | 2.050^***^ | 2.050^***^ | 2.049^***^ | 2.052^***^ |
|  | (0.041) | (0.041) | (0.041) | (0.041) | (0.041) | (0.042) | (0.041) | (0.040) | (0.042) |
| High School | 0.584^***^ | 0.584^***^ | 0.584^***^ | 0.584^***^ | 0.584^***^ | 0.584^***^ | 0.584^***^ | 0.584^***^ | 0.584^***^ |
|  | (0.015) | (0.015) | (0.015) | (0.015) | (0.015) | (0.015) | (0.015) | (0.015) | (0.015) |
| Employment (ref.=Employed) |  |  |  |  |  |  |  |  |  |
| Unemployed | 3.670^***^ | 3.670^***^ | 3.670^***^ | 3.670^***^ | 3.670^***^ | 3.670^***^ | 3.671^***^ | 3.670^***^ | 3.671^***^ |
|  | (0.107) | (0.107) | (0.107) | (0.107) | (0.107) | (0.107) | (0.107) | (0.107) | (0.107) |
| Not in Labor Force | 9.617^***^ | 9.616^***^ | 9.617^***^ | 9.617^***^ | 9.616^***^ | 9.616^***^ | 9.618^***^ | 9.617^***^ | 9.617^***^ |
|  | (0.383) | (0.382) | (0.383) | (0.382) | (0.382) | (0.381) | (0.382) | (0.382) | (0.382) |
| Married | 0.445^***^ | 0.445^***^ | 0.445^***^ | 0.445^***^ | 0.445^***^ | 0.445^***^ | 0.445^***^ | 0.445^***^ | 0.445^***^ |
|  | (0.004) | (0.004) | (0.004) | (0.004) | (0.004) | (0.004) | (0.004) | (0.004) | (0.004) |
| Number of Children | 0.837^***^ | 0.837^***^ | 0.837^***^ | 0.837^***^ | 0.837^***^ | 0.837^***^ | 0.837^***^ | 0.837^***^ | 0.837^***^ |
|  | (0.011) | (0.011) | (0.011) | (0.011) | (0.011) | (0.011) | (0.011) | (0.011) | (0.011) |
| Not in Metro | 1.027 | 1.027 | 1.027 | 1.027 | 1.027 | 1.027 | 1.026 | 1.026 | 1.026 |
|  | (0.030) | (0.030) | (0.030) | (0.030) | (0.030) | (0.030) | (0.030) | (0.030) | (0.030) |
| Moved in Past Year | 0.862^**^ | 0.862^**^ | 0.862^**^ | 0.862^***^ | 0.862^***^ | 0.861^***^ | 0.861^**^ | 0.861^**^ | 0.861^***^ |
|  | (0.039) | (0.039) | (0.039) | (0.039) | (0.039) | (0.039) | (0.039) | (0.039) | (0.039) |
| State-Level |  |  |  |  |  |  |  |  |  |
| % Latino | 0.937^**^ | 0.932^***^ | 0.936^**^ | 0.937^**^ | 0.932^***^ | 0.936^**^ | 0.938^**^ | 0.932^***^ | 0.936^**^ |
|  | (0.019) | (0.020) | (0.020) | (0.019) | (0.019) | (0.020) | (0.019) | (0.020) | (0.020) |
| % Immigrant | 1.048^*^ | 1.051^**^ | 1.056^**^ | 1.048^*^ | 1.051^**^ | 1.057^**^ | 1.048^*^ | 1.051^**^ | 1.057^**^ |
|  | (0.019) | (0.020) | (0.021) | (0.019) | (0.020) | (0.021) | (0.019) | (0.020) | (0.021) |
| Expanded Medicaid | 1.099^**^ | 1.067^*^ | 1.074^*^ | 1.099^**^ | 1.067^*^ | 1.074^*^ | 1.099^**^ | 1.066^*^ | 1.074^*^ |
|  | (0.037) | (0.030) | (0.038) | (0.037) | (0.030) | (0.038) | (0.037) | (0.030) | (0.038) |
| Unemployment Rate | 1.000 | 1.005 | 0.998 | 1.000 | 1.005 | 0.998 | 1.000 | 1.005 | 0.998 |
|  | (0.008) | (0.010) | (0.007) | (0.008) | (0.010) | (0.007) | (0.008) | (0.010) | (0.007) |
| % with BA | 1.011 | 1.008 | 1.005 | 1.011 | 1.008 | 1.004 | 1.011 | 1.008 | 1.004 |
|  | (0.022) | (0.023) | (0.023) | (0.022) | (0.023) | (0.023) | (0.022) | (0.023) | (0.023) |
| Constant | 0.360 | 0.409 | 0.383 | 0.360 | 0.408 | 0.382 | 0.359 | 0.407 | 0.382 |
|  | (0.274) | (0.352) | (0.318) | (0.274) | (0.349) | (0.318) | (0.274) | (0.350) | (0.318) |
| Pseudo *R*^2^ | 0.189 | 0.189 | 0.189 | 0.189 | 0.189 | 0.189 | 0.190 | 0.189 | 0.189 |

*Note*. FB=foreign-born; models adjust for state and year fixed effects and cluster standard errors; ^*^*p* < .05, ^**^*p* < .01, ^***^*p* < .001

**Table S7.** *Odds Ratios (Standard Errors) from Fixed Effects Models of Associations Between Policy Liberalization Scores and Ambulatory Difficulty (2-year lag) among Pan-Ethnic Latino Adults Aged 45–67 Years, by Nativity and Citizenship*

|  | Model 1 | Model 2 | Model 3 | Model 4 | Model 5 | Model 6 | Model 7 | Model 8 | Model 9 |
| --- | --- | --- | --- | --- | --- | --- | --- | --- | --- |
| Policy |  |  |  |  |  |  |  |  |  |
| Overall | 0.805 |  |  | 0.707^**^ |  |  | 0.711^**^ |  |  |
|  | (0.101) |  |  | (0.087) |  |  | (0.089) |  |  |
| Economic |  | 0.734^***^ |  |  | 0.641^***^ |  |  | 0.651^***^ |  |
|  |  | (0.048) |  |  | (0.043) |  |  | (0.043) |  |
| Social |  |  | 0.920 |  |  | 0.811^*^ |  |  | 0.819 |
|  |  |  | (0.100) |  |  | (0.083) |  |  | (0.088) |
| FB | 0.900^***^ | 0.901^***^ | 0.900^***^ | 0.781^***^ | 0.790^***^ | 0.793^***^ | 0.899^***^ | 0.900^***^ | 0.898^***^ |
|  | (0.016) | (0.016) | (0.016) | (0.020) | (0.022) | (0.019) | (0.014) | (0.014) | (0.014) |
| FB × Policy |  |  |  |  |  |  |  |  |  |
| FB × Overall |  |  |  | 1.304^***^ |  |  |  |  |  |
|  |  |  |  | (0.032) |  |  |  |  |  |
| FB × Economic |  |  |  |  | 1.270^***^ |  |  |  |  |
|  |  |  |  |  | (0.030) |  |  |  |  |
| FB × Social |  |  |  |  |  | 1.284^***^ |  |  |  |
|  |  |  |  |  |  | (0.037) |  |  |  |
| Citizenship (ref.=Citizen) |  |  |  |  |  |  |  |  |  |
| Naturalized | 0.781^***^ | 0.781^***^ | 0.781^***^ | 0.788^***^ | 0.794^***^ | 0.780^***^ | 0.677^***^ | 0.689^***^ | 0.683^***^ |
|  | (0.026) | (0.026) | (0.026) | (0.019) | (0.022) | (0.017) | (0.011) | (0.012) | (0.011) |
| Non-Citizen | 0.677^***^ | 0.677^***^ | 0.677^***^ | 0.686^***^ | 0.691^***^ | 0.679^***^ | 0.596^***^ | 0.606^***^ | 0.598^***^ |
|  | (0.030) | (0.030) | (0.030) | (0.019) | (0.021) | (0.018) | (0.017) | (0.017) | (0.017) |
| Citizenship × Policy |  |  |  |  |  |  |  |  |  |
| Naturalized × Overall |  |  |  |  |  |  | 1.325^***^ |  |  |
|  |  |  |  |  |  |  | (0.035) |  |  |
| Non-Citizen × Overall |  |  |  |  |  |  | 1.302^***^ |  |  |
|  |  |  |  |  |  |  | (0.039) |  |  |
| Naturalized × Economic |  |  |  |  |  |  |  | 1.296^***^ |  |
|  |  |  |  |  |  |  |  | (0.027) |  |
| Non-Citizen × Economic |  |  |  |  |  |  |  | 1.269^***^ |  |
|  |  |  |  |  |  |  |  | (0.037) |  |
| Naturalized × Social |  |  |  |  |  |  |  |  | 1.302^***^ |
|  |  |  |  |  |  |  |  |  | (0.038) |
| Non-Citizen × Social |  |  |  |  |  |  |  |  | 1.286^***^ |
|  |  |  |  |  |  |  |  |  | (0.039) |
| Country of Origin (ref.=Mexico) |  |  |  |  |  |  |  |  |  |
| Puerto Rico | 1.258^***^ | 1.258^***^ | 1.258^***^ | 1.273^***^ | 1.280^***^ | 1.264^***^ | 1.268^***^ | 1.275^***^ | 1.260^***^ |
|  | (0.055) | (0.055) | (0.055) | (0.037) | (0.037) | (0.038) | (0.036) | (0.036) | (0.037) |
| Cuba | 1.005 | 1.006 | 1.005 | 1.014 | 1.015 | 1.014 | 1.025 | 1.028 | 1.023 |
|  | (0.024) | (0.024) | (0.024) | (0.025) | (0.024) | (0.025) | (0.027) | (0.027) | (0.027) |
| Other | 1.007 | 1.007 | 1.007 | 1.005 | 1.004 | 1.007 | 1.002 | 1.000 | 1.005 |
|  | (0.021) | (0.021) | (0.021) | (0.020) | (0.020) | (0.020) | (0.021) | (0.020) | (0.021) |
| Age | 1.024^***^ | 1.024^***^ | 1.024^***^ | 1.023^***^ | 1.023^***^ | 1.023^***^ | 1.023^***^ | 1.023^***^ | 1.023^***^ |
|  | (0.002) | (0.002) | (0.002) | (0.002) | (0.002) | (0.002) | (0.002) | (0.002) | (0.002) |
| Female | 0.902^**^ | 0.902^**^ | 0.902^**^ | 0.902^**^ | 0.902^**^ | 0.902^**^ | 0.902^**^ | 0.902^**^ | 0.902^**^ |
|  | (0.034) | (0.034) | (0.034) | (0.034) | (0.034) | (0.034) | (0.035) | (0.035) | (0.035) |
| Health Insurance | 1.954^***^ | 1.954^***^ | 1.954^***^ | 1.948^***^ | 1.948^***^ | 1.948^***^ | 1.947^***^ | 1.947^***^ | 1.947^***^ |
|  | (0.050) | (0.050) | (0.050) | (0.049) | (0.049) | (0.049) | (0.049) | (0.049) | (0.049) |
| High School | 0.687^***^ | 0.687^***^ | 0.687^***^ | 0.690^***^ | 0.689^***^ | 0.690^***^ | 0.689^***^ | 0.689^***^ | 0.689^***^ |
|  | (0.013) | (0.013) | (0.013) | (0.013) | (0.013) | (0.013) | (0.014) | (0.013) | (0.013) |
| Employment (ref.=Employed) |  |  |  |  |  |  |  |  |  |
| Unemployed | 2.639^***^ | 2.639^***^ | 2.639^***^ | 2.640^***^ | 2.640^***^ | 2.640^***^ | 2.639^***^ | 2.639^***^ | 2.639^***^ |
|  | (0.106) | (0.106) | (0.106) | (0.107) | (0.107) | (0.107) | (0.106) | (0.107) | (0.106) |
| Not in Labor Force | 7.396^***^ | 7.396^***^ | 7.396^***^ | 7.401^***^ | 7.400^***^ | 7.401^***^ | 7.403^***^ | 7.402^***^ | 7.403^***^ |
|  | (0.124) | (0.124) | (0.124) | (0.125) | (0.125) | (0.124) | (0.125) | (0.125) | (0.124) |
| Married | 0.571^***^ | 0.571^***^ | 0.571^***^ | 0.571^***^ | 0.571^***^ | 0.571^***^ | 0.570^***^ | 0.570^***^ | 0.570^***^ |
|  | (0.005) | (0.005) | (0.005) | (0.005) | (0.005) | (0.005) | (0.005) | (0.005) | (0.005) |
| Number of Children | 0.937^***^ | 0.937^***^ | 0.937^***^ | 0.935^***^ | 0.936^***^ | 0.935^***^ | 0.935^***^ | 0.935^***^ | 0.935^***^ |
|  | (0.004) | (0.004) | (0.004) | (0.004) | (0.004) | (0.004) | (0.004) | (0.004) | (0.004) |
| Not in Metro | 1.105^***^ | 1.105^***^ | 1.105^***^ | 1.100^***^ | 1.101^***^ | 1.100^***^ | 1.100^***^ | 1.101^***^ | 1.100^***^ |
|  | (0.028) | (0.028) | (0.028) | (0.027) | (0.027) | (0.028) | (0.027) | (0.027) | (0.028) |
| Moved in Past Year | 0.899^*^ | 0.899^*^ | 0.899^*^ | 0.901 | 0.901 | 0.902 | 0.899^*^ | 0.899^*^ | 0.900^*^ |
|  | (0.048) | (0.048) | (0.048) | (0.048) | (0.048) | (0.048) | (0.048) | (0.048) | (0.048) |
| State-Level |  |  |  |  |  |  |  |  |  |
| % Latino | 0.972 | 0.972 | 0.970 | 0.973 | 0.973 | 0.971 | 0.973 | 0.973 | 0.971 |
|  | (0.019) | (0.017) | (0.021) | (0.020) | (0.017) | (0.021) | (0.020) | (0.017) | (0.021) |
| % Immigrant | 1.059^***^ | 1.050^***^ | 1.064^***^ | 1.058^***^ | 1.050^***^ | 1.063^***^ | 1.058^***^ | 1.050^***^ | 1.064^***^ |
|  | (0.015) | (0.015) | (0.016) | (0.015) | (0.015) | (0.016) | (0.016) | (0.015) | (0.016) |
| Expanded Medicaid | 1.079^**^ | 1.082^**^ | 1.065^**^ | 1.080^**^ | 1.085^**^ | 1.066^**^ | 1.079^**^ | 1.082^**^ | 1.065^**^ |
|  | (0.027) | (0.029) | (0.026) | (0.028) | (0.030) | (0.026) | (0.028) | (0.030) | (0.026) |
| Unemployment Rate | 0.999 | 0.999 | 1.000 | 1.000 | 1.000 | 1.001 | 1.000 | 1.000 | 1.001 |
|  | (0.011) | (0.011) | (0.011) | (0.012) | (0.011) | (0.011) | (0.012) | (0.011) | (0.012) |
| % with BA | 0.964 | 0.968 | 0.960^*^ | 0.965 | 0.970 | 0.961 | 0.964 | 0.969 | 0.961 |
|  | (0.019) | (0.018) | (0.020) | (0.019) | (0.018) | (0.020) | (0.019) | (0.018) | (0.020) |
| Constant | 0.0498^***^ | 0.0497^***^ | 0.0526^***^ | 0.0497^***^ | 0.0479^***^ | 0.0532^***^ | 0.0498^***^ | 0.0489^***^ | 0.0526^***^ |
|  | (0.035) | (0.033) | (0.040) | (0.035) | (0.031) | (0.040) | (0.035) | (0.032) | (0.040) |
| Pseudo *R*^2^ | 0.178 | 0.178 | 0.178 | 0.178 | 0.178 | 0.178 | 0.178 | 0.178 | 0.178 |

*Note*. FB=foreign-born; models adjust for state and year fixed effects and cluster standard errors; ^*^*p* < .05, ^**^*p* < .01, ^***^*p* < .001

**Table S8.** *Odds Ratios (Standard Errors) from Fixed Effects Models of Associations Between Policy Liberalization Scores and Independent Living Difficulty (2-year lag) among Pan-Ethnic Latino Adults Aged 45–67 Years, by Nativity and Citizenship*

|  | Model 1 | Model 2 | Model 3 | Model 4 | Model 5 | Model 6 | Model 7 | Model 8 | Model 9 |
| --- | --- | --- | --- | --- | --- | --- | --- | --- | --- |
| Policy |  |  |  |  |  |  |  |  |  |
| Overall | 0.832 |  |  | 0.796 |  |  | 0.783 |  |  |
|  | (0.140) |  |  | (0.146) |  |  | (0.141) |  |  |
| Economic |  | 0.763^***^ |  |  | 0.738^***^ |  |  | 0.723^***^ |  |
|  |  | (0.059) |  |  | (0.067) |  |  | (0.058) |  |
| Social |  |  | 0.909 |  |  | 0.866 |  |  | 0.864 |
|  |  |  | (0.149) |  |  | (0.152) |  |  | (0.152) |
| FB | 0.900^***^ | 0.900^***^ | 0.900^***^ | 0.855^***^ | 0.869^***^ | 0.855^***^ | 0.899^***^ | 0.900^***^ | 0.899^***^ |
|  | (0.027) | (0.027) | (0.027) | (0.027) | (0.025) | (0.028) | (0.026) | (0.026) | (0.026) |
| FB × Policy |  |  |  |  |  |  |  |  |  |
| FB × Overall |  |  |  | 1.098^*^ |  |  |  |  |  |
|  |  |  |  | (0.049) |  |  |  |  |  |
| FB × Economic |  |  |  |  | 1.064 |  |  |  |  |
|  |  |  |  |  | (0.051) |  |  |  |  |
| FB × Social |  |  |  |  |  | 1.105^*^ |  |  |  |
|  |  |  |  |  |  | (0.043) |  |  |  |
| Citizenship (ref.=Citizen) |  |  |  |  |  |  |  |  |  |
| Naturalized | 0.743^***^ | 0.742^***^ | 0.743^***^ | 0.745^***^ | 0.746^***^ | 0.742^***^ | 0.675^***^ | 0.684^***^ | 0.680^***^ |
|  | (0.021) | (0.021) | (0.021) | (0.020) | (0.020) | (0.020) | (0.023) | (0.022) | (0.022) |
| Non-Citizen | 0.692^***^ | 0.692^***^ | 0.692^***^ | 0.695^***^ | 0.695^***^ | 0.692^***^ | 0.669^***^ | 0.673^***^ | 0.671^***^ |
|  | (0.020) | (0.020) | (0.020) | (0.020) | (0.020) | (0.019) | (0.029) | (0.028) | (0.027) |
| Citizenship × Policy |  |  |  |  |  |  |  |  |  |
| Naturalized × Overall |  |  |  |  |  |  | 1.198^***^ |  |  |
|  |  |  |  |  |  |  | (0.045) |  |  |
| Non-Citizen × Overall |  |  |  |  |  |  | 1.073 |  |  |
|  |  |  |  |  |  |  | (0.054) |  |  |
| Naturalized × Economic |  |  |  |  |  |  |  | 1.176^***^ |  |
|  |  |  |  |  |  |  |  | (0.039) |  |
| Non-Citizen × Economic |  |  |  |  |  |  |  | 1.062 |  |
|  |  |  |  |  |  |  |  | (0.052) |  |
| Naturalized × Social |  |  |  |  |  |  |  |  | 1.180^***^ |
|  |  |  |  |  |  |  |  |  | (0.043) |
| Non-Citizen × Social |  |  |  |  |  |  |  |  | 1.066 |
|  |  |  |  |  |  |  |  |  | (0.048) |
| Country of Origin (ref.=Mexico) |  |  |  |  |  |  |  |  |  |
| Puerto Rico | 1.198^***^ | 1.199^***^ | 1.198^***^ | 1.203^***^ | 1.204^***^ | 1.201^***^ | 1.202^***^ | 1.205^***^ | 1.198^***^ |
|  | (0.050) | (0.049) | (0.050) | (0.045) | (0.046) | (0.045) | (0.043) | (0.044) | (0.044) |
| Cuba | 1.327^***^ | 1.327^***^ | 1.327^***^ | 1.331^***^ | 1.330^***^ | 1.331^***^ | 1.341^***^ | 1.343^***^ | 1.340^***^ |
|  | (0.053) | (0.052) | (0.052) | (0.054) | (0.054) | (0.054) | (0.059) | (0.060) | (0.058) |
| Other | 1.065^*^ | 1.065^*^ | 1.065^*^ | 1.064^*^ | 1.064^*^ | 1.064^*^ | 1.062^*^ | 1.061^*^ | 1.063^*^ |
|  | (0.032) | (0.032) | (0.032) | (0.032) | (0.032) | (0.032) | (0.032) | (0.031) | (0.032) |
| Age | 0.992^***^ | 0.992^***^ | 0.992^***^ | 0.992^***^ | 0.992^***^ | 0.992^***^ | 0.992^***^ | 0.992^***^ | 0.992^***^ |
|  | (0.002) | (0.002) | (0.002) | (0.002) | (0.002) | (0.002) | (0.002) | (0.002) | (0.002) |
| Female | 0.882^**^ | 0.882^**^ | 0.882^**^ | 0.882^**^ | 0.882^**^ | 0.882^**^ | 0.882^**^ | 0.882^**^ | 0.882^**^ |
|  | (0.036) | (0.036) | (0.036) | (0.036) | (0.036) | (0.036) | (0.036) | (0.036) | (0.036) |
| Health Insurance | 2.734^***^ | 2.734^***^ | 2.734^***^ | 2.731^***^ | 2.732^***^ | 2.731^***^ | 2.733^***^ | 2.733^***^ | 2.733^***^ |
|  | (0.090) | (0.090) | (0.090) | (0.088) | (0.088) | (0.088) | (0.090) | (0.089) | (0.089) |
| High School | 0.633^***^ | 0.633^***^ | 0.633^***^ | 0.633^***^ | 0.633^***^ | 0.634^***^ | 0.633^***^ | 0.633^***^ | 0.633^***^ |
|  | (0.008) | (0.008) | (0.008) | (0.009) | (0.009) | (0.009) | (0.009) | (0.009) | (0.009) |
| Employment (ref.=Employed) |  |  |  |  |  |  |  |  |  |
| Unemployed | 3.589^***^ | 3.590^***^ | 3.589^***^ | 3.590^***^ | 3.590^***^ | 3.590^***^ | 3.590^***^ | 3.591^***^ | 3.590^***^ |
|  | (0.165) | (0.165) | (0.165) | (0.166) | (0.166) | (0.166) | (0.166) | (0.166) | (0.166) |
| Not in Labor Force | 15.90^***^ | 15.90^***^ | 15.90^***^ | 15.90^***^ | 15.90^***^ | 15.90^***^ | 15.90^***^ | 15.90^***^ | 15.90^***^ |
|  | (0.391) | (0.390) | (0.391) | (0.392) | (0.392) | (0.392) | (0.393) | (0.393) | (0.392) |
| Married | 0.508^***^ | 0.508^***^ | 0.508^***^ | 0.508^***^ | 0.508^***^ | 0.508^***^ | 0.508^***^ | 0.508^***^ | 0.508^***^ |
|  | (0.009) | (0.009) | (0.009) | (0.009) | (0.009) | (0.009) | (0.009) | (0.009) | (0.009) |
| Number of Children | 0.884^***^ | 0.884^***^ | 0.884^***^ | 0.884^***^ | 0.884^***^ | 0.884^***^ | 0.883^***^ | 0.883^***^ | 0.883^***^ |
|  | (0.008) | (0.008) | (0.008) | (0.008) | (0.008) | (0.008) | (0.008) | (0.008) | (0.008) |
| Not in Metro | 0.997 | 0.997 | 0.997 | 0.995 | 0.996 | 0.995 | 0.994 | 0.995 | 0.994 |
|  | (0.041) | (0.041) | (0.041) | (0.042) | (0.042) | (0.042) | (0.042) | (0.042) | (0.042) |
| Moved in Past Year | 0.868^*^ | 0.868^*^ | 0.868^*^ | 0.869^*^ | 0.869^*^ | 0.869^*^ | 0.868^*^ | 0.867^*^ | 0.868^*^ |
|  | (0.054) | (0.054) | (0.054) | (0.054) | (0.054) | (0.054) | (0.054) | (0.054) | (0.054) |
| State-Level |  |  |  |  |  |  |  |  |  |
| % Latino | 0.949^***^ | 0.949^***^ | 0.948^***^ | 0.949^***^ | 0.949^***^ | 0.948^***^ | 0.950^***^ | 0.950^***^ | 0.948^**^ |
|  | (0.014) | (0.013) | (0.015) | (0.014) | (0.013) | (0.015) | (0.014) | (0.013) | (0.015) |
| % Immigrant | 1.057^***^ | 1.050^***^ | 1.060^***^ | 1.057^***^ | 1.049^***^ | 1.060^***^ | 1.057^***^ | 1.049^***^ | 1.061^***^ |
|  | (0.013) | (0.013) | (0.015) | (0.013) | (0.013) | (0.015) | (0.013) | (0.013) | (0.015) |
| Expanded Medicaid | 1.076^**^ | 1.080^***^ | 1.068^*^ | 1.077^**^ | 1.081^***^ | 1.068^*^ | 1.077^**^ | 1.080^***^ | 1.067^*^ |
|  | (0.029) | (0.024) | (0.031) | (0.029) | (0.024) | (0.031) | (0.029) | (0.024) | (0.030) |
| Unemployment Rate | 1.014 | 1.014 | 1.014 | 1.014 | 1.014 | 1.014 | 1.014 | 1.014 | 1.014 |
|  | (0.010) | (0.010) | (0.010) | (0.010) | (0.010) | (0.010) | (0.010) | (0.010) | (0.010) |
| % with BA | 0.995 | 0.998 | 0.992 | 0.995 | 0.999 | 0.993 | 0.995 | 0.999 | 0.992 |
|  | (0.020) | (0.018) | (0.021) | (0.019) | (0.018) | (0.021) | (0.020) | (0.018) | (0.021) |
| Constant | 0.047^***^ | 0.048^***^ | 0.049^***^ | 0.048^***^ | 0.047^***^ | 0.050^***^ | 0.048^***^ | 0.047^***^ | 0.049^***^ |
|  | (0.033) | (0.031) | (0.035) | (0.033) | (0.030) | (0.036) | (0.032) | (0.030) | (0.035) |
| Pseudo *R*^2^ | 0.223 | 0.223 | 0.223 | 0.223 | 0.223 | 0.223 | 0.223 | 0.223 | 0.223 |

*Note*. FB=foreign-born; models adjust for state and year fixed effects and cluster standard errors; ^*^*p* < .05, ^**^*p* < .01, ^***^*p* < .001

**Table S9.** *Odds Ratios (Standard Errors) from Fixed Effects Models of Associations Between Policy Liberalization Scores and Self-Care Difficulty (2-year lag) among Pan-Ethnic Latino Adults Aged 45–67 Years, by Nativity and Citizenship*

|  | Model 1 | Model 2 | Model 3 | Model 4 | Model 5 | Model 6 | Model 7 | Model 8 | Model 9 |
| --- | --- | --- | --- | --- | --- | --- | --- | --- | --- |
| Policy |  |  |  |  |  |  |  |  |  |
| Overall | 0.955 |  |  | 0.880 |  |  | 0.855 |  |  |
|  | (0.171) |  |  | (0.162) |  |  | (0.152) |  |  |
| Economic |  | 0.766^*^ |  |  | 0.714^**^ |  |  | 0.690^***^ |  |
|  |  | (0.084) |  |  | (0.080) |  |  | (0.068) |  |
| Social |  |  | 1.122 |  |  | 1.029 |  |  | 1.018 |
|  |  |  | (0.203) |  |  | (0.191) |  |  | (0.192) |
| FB | 0.971 | 0.972 | 0.971 | 0.886^*^ | 0.904^*^ | 0.888^*^ | 0.971 | 0.972 | 0.970 |
|  | (0.054) | (0.055) | (0.054) | (0.046) | (0.044) | (0.048) | (0.052) | (0.052) | (0.051) |
| FB × Policy |  |  |  |  |  |  |  |  |  |
| FB × Overall |  |  |  | 1.184^***^ |  |  |  |  |  |
|  |  |  |  | (0.059) |  |  |  |  |  |
| FB × Economic |  |  |  |  | 1.137^*^ |  |  |  |  |
|  |  |  |  |  | (0.065) |  |  |  |  |
| FB × Social |  |  |  |  |  | 1.191^***^ |  |  |  |
|  |  |  |  |  |  | (0.044) |  |  |  |
| Citizenship (ref.=Citizen) |  |  |  |  |  |  |  |  |  |
| Naturalized | 0.804^***^ | 0.803^***^ | 0.804^***^ | 0.809^***^ | 0.811^***^ | 0.804^***^ | 0.679^***^ | 0.693^***^ | 0.686^***^ |
|  | (0.048) | (0.048) | (0.048) | (0.050) | (0.050) | (0.048) | (0.042) | (0.040) | (0.042) |
| Non-Citizen | 0.680^***^ | 0.680^***^ | 0.680^***^ | 0.686^***^ | 0.688^***^ | 0.681^***^ | 0.640^***^ | 0.643^***^ | 0.643^***^ |
|  | (0.037) | (0.037) | (0.037) | (0.041) | (0.041) | (0.040) | (0.055) | (0.054) | (0.053) |
| Citizenship × Policy |  |  |  |  |  |  |  |  |  |
| Naturalized × Overall |  |  |  |  |  |  | 1.376^***^ |  |  |
|  |  |  |  |  |  |  | (0.074) |  |  |
| Non-Citizen × Overall |  |  |  |  |  |  | 1.139 |  |  |
|  |  |  |  |  |  |  | (0.094) |  |  |
| Naturalized × Economic |  |  |  |  |  |  |  | 1.339^***^ |  |
|  |  |  |  |  |  |  |  | (0.064) |  |
| Non-Citizen × Economic |  |  |  |  |  |  |  | 1.131 |  |
|  |  |  |  |  |  |  |  | (0.094) |  |
| Naturalized × Social |  |  |  |  |  |  |  |  | 1.348^***^ |
|  |  |  |  |  |  |  |  |  | (0.077) |
| Non-Citizen × Social |  |  |  |  |  |  |  |  | 1.127 |
|  |  |  |  |  |  |  |  |  | (0.086) |
| Country of Origin (ref.=Mexico) |  |  |  |  |  |  |  |  |  |
| Puerto Rico | 1.160^**^ | 1.160^**^ | 1.160^**^ | 1.169^***^ | 1.171^***^ | 1.164^***^ | 1.166^***^ | 1.172^***^ | 1.160^***^ |
|  | (0.053) | (0.053) | (0.053) | (0.043) | (0.046) | (0.042) | (0.038) | (0.039) | (0.039) |
| Cuba | 1.170^**^ | 1.170^**^ | 1.170^**^ | 1.177^**^ | 1.176^**^ | 1.177^**^ | 1.193^**^ | 1.196^***^ | 1.191^**^ |
|  | (0.057) | (0.057) | (0.057) | (0.060) | (0.060) | (0.060) | (0.064) | (0.064) | (0.064) |
| Other | 1.060 | 1.060 | 1.060 | 1.059 | 1.058 | 1.059 | 1.055 | 1.053 | 1.057 |
|  | (0.038) | (0.038) | (0.038) | (0.038) | (0.038) | (0.038) | (0.038) | (0.037) | (0.039) |
| Age | 0.993^***^ | 0.993^***^ | 0.993^***^ | 0.993^***^ | 0.993^***^ | 0.993^***^ | 0.993^***^ | 0.993^***^ | 0.993^***^ |
|  | (0.002) | (0.002) | (0.002) | (0.002) | (0.001) | (0.002) | (0.002) | (0.002) | (0.002) |
| Female | 0.758^***^ | 0.758^***^ | 0.758^***^ | 0.757^***^ | 0.757^***^ | 0.757^***^ | 0.758^***^ | 0.758^***^ | 0.758^***^ |
|  | (0.026) | (0.026) | (0.026) | (0.026) | (0.026) | (0.026) | (0.026) | (0.026) | (0.026) |
| Health Insurance | 2.784^***^ | 2.785^***^ | 2.784^***^ | 2.778^***^ | 2.780^***^ | 2.777^***^ | 2.781^***^ | 2.782^***^ | 2.781^***^ |
|  | (0.126) | (0.125) | (0.126) | (0.123) | (0.123) | (0.123) | (0.126) | (0.127) | (0.126) |
| High School | 0.678^***^ | 0.678^***^ | 0.678^***^ | 0.679^***^ | 0.679^***^ | 0.679^***^ | 0.679^***^ | 0.679^***^ | 0.679^***^ |
|  | (0.012) | (0.012) | (0.012) | (0.013) | (0.012) | (0.013) | (0.013) | (0.013) | (0.013) |
| Employment (ref.=Employed) |  |  |  |  |  |  |  |  |  |
| Unemployed | 3.269^***^ | 3.269^***^ | 3.268^***^ | 3.269^***^ | 3.270^***^ | 3.268^***^ | 3.270^***^ | 3.270^***^ | 3.269^***^ |
|  | (0.236) | (0.236) | (0.236) | (0.236) | (0.236) | (0.236) | (0.236) | (0.236) | (0.236) |
| Not in Labor Force | 14.57^***^ | 14.57^***^ | 14.57^***^ | 14.57^***^ | 14.57^***^ | 14.57^***^ | 14.58^***^ | 14.58^***^ | 14.58^***^ |
|  | (0.476) | (0.476) | (0.476) | (0.477) | (0.477) | (0.477) | (0.476) | (0.477) | (0.476) |
| Married | 0.553^***^ | 0.553^***^ | 0.553^***^ | 0.553^***^ | 0.553^***^ | 0.553^***^ | 0.552^***^ | 0.552^***^ | 0.552^***^ |
|  | (0.009) | (0.009) | (0.009) | (0.009) | (0.009) | (0.009) | (0.009) | (0.009) | (0.009) |
| Number of Children | 0.898^***^ | 0.898^***^ | 0.898^***^ | 0.897^***^ | 0.897^***^ | 0.897^***^ | 0.897^***^ | 0.897^***^ | 0.897^***^ |
|  | (0.012) | (0.012) | (0.012) | (0.012) | (0.012) | (0.012) | (0.012) | (0.012) | (0.012) |
| Not in Metro | 1.011 | 1.011 | 1.011 | 1.008 | 1.009 | 1.008 | 1.007 | 1.007 | 1.007 |
|  | (0.028) | (0.028) | (0.028) | (0.029) | (0.029) | (0.029) | (0.029) | (0.029) | (0.029) |
| Moved in Past Year | 0.840^*^ | 0.840^*^ | 0.840^*^ | 0.841^*^ | 0.841^*^ | 0.842^*^ | 0.839^*^ | 0.838^*^ | 0.839^*^ |
|  | (0.059) | (0.059) | (0.059) | (0.059) | (0.059) | (0.059) | (0.059) | (0.059) | (0.059) |
| State-Level |  |  |  |  |  |  |  |  |  |
| % Latino | 0.969 | 0.971 | 0.965 | 0.970 | 0.972 | 0.966 | 0.971 | 0.973 | 0.966 |
|  | (0.018) | (0.017) | (0.019) | (0.018) | (0.017) | (0.019) | (0.018) | (0.017) | (0.019) |
| % Immigrant | 1.064^**^ | 1.051^*^ | 1.071^**^ | 1.064^**^ | 1.051^*^ | 1.071^**^ | 1.064^**^ | 1.051^*^ | 1.072^**^ |
|  | (0.024) | (0.023) | (0.024) | (0.024) | (0.023) | (0.024) | (0.024) | (0.024) | (0.024) |
| Expanded Medicaid | 1.065 | 1.083^*^ | 1.047 | 1.066 | 1.085^*^ | 1.047 | 1.065 | 1.082^*^ | 1.047 |
|  | (0.041) | (0.042) | (0.041) | (0.041) | (0.042) | (0.041) | (0.041) | (0.042) | (0.041) |
| Unemployment Rate | 1.022 | 1.020 | 1.027 | 1.023 | 1.020 | 1.027 | 1.022 | 1.020 | 1.027 |
|  | (0.016) | (0.015) | (0.016) | (0.016) | (0.015) | (0.016) | (0.016) | (0.015) | (0.016) |
| % with BA | 1.007 | 1.014 | 1.003 | 1.007 | 1.015 | 1.003 | 1.007 | 1.014 | 1.003 |
|  | (0.027) | (0.026) | (0.027) | (0.027) | (0.026) | (0.027) | (0.028) | (0.027) | (0.028) |
| Constant | 0.009^***^ | 0.008^***^ | 0.009^***^ | 0.008^***^ | 0.008^***^ | 0.009^***^ | 0.008^***^ | 0.008^***^ | 0.009^***^ |
|  | (0.007) | (0.007) | (0.008) | (0.007) | (0.007) | (0.008) | (0.007) | (0.007) | (0.008) |
| Pseudo *R*^2^ | 0.190 | 0.190 | 0.190 | 0.190 | 0.190 | 0.190 | 0.190 | 0.190 | 0.190 |

*Note*. FB=foreign-born; models adjust for state and year fixed effects and cluster standard errors; ^*^*p* < .05, ^**^*p* < .01, ^***^*p* < .001
